# Molecular characterization of colorectal cancer related peritoneal metastatic disease

**DOI:** 10.1038/s41467-022-32198-z

**Published:** 2022-08-04

**Authors:** Kristiaan J. Lenos, Sander Bach, Leandro Ferreira Moreno, Sanne ten Hoorn, Nina R. Sluiter, Sanne Bootsma, Felipe A. Vieira Braga, Lisanne E. Nijman, Tom van den Bosch, Daniel M. Miedema, Erik van Dijk, Bauke Ylstra, Ruth Kulicke, Fred P. Davis, Nicolas Stransky, Gromoslaw A. Smolen, Robert R. J. Coebergh van den Braak, Jan N. M. IJzermans, John W. M. Martens, Sally Hallam, Andrew D. Beggs, Geert J. P. L. Kops, Nico Lansu, Vivian P. Bastiaenen, Charlotte E. L. Klaver, Maria C. Lecca, Khalid El Makrini, Clara C. Elbers, Mark P. G. Dings, Carel J. M. van Noesel, Onno Kranenburg, Jan Paul Medema, Jan Koster, Lianne Koens, Cornelis J. A. Punt, Pieter J. Tanis, Ignace H. de Hingh, Maarten F. Bijlsma, Jurriaan B. Tuynman, Louis Vermeulen

**Affiliations:** 1 Amsterdam UMC location University of Amsterdam, Center for Experimental and Molecular Medicine, Laboratory for Experimental Oncology and Radiobiology, Cancer Center Amsterdam, Amsterdam Gastroenterology Endocrinology Metabolism, Meibergdreef 9, Amsterdam, The Netherlands; 2 Oncode Institute, Amsterdam, The Netherlands; 3 Amsterdam UMC location Vrije Universiteit Amsterdam, Department of Surgery, Cancer Center Amsterdam, De Boelelaan 1117, Amsterdam, The Netherlands; 4 Amsterdam UMC location Vrije Universiteit Amsterdam, Department of Pathology, Cancer Center Amsterdam, De Boelelaan 1117, Amsterdam, The Netherlands; 5 Celsius Therapeutics, 399 Binney Street, Cambridge, MA 02139 USA; 6 Department of Surgery, Erasmus MC University Medical Center, Rotterdam, The Netherlands; 7 Department of Medical Oncology, Erasmus MC Cancer Institute, Erasmus MC University Medical Center, Rotterdam, the Netherlands & Cancer Genomics Center, Utrecht, The Netherlands; 8 Surgical Research Laboratory, Institute of Cancer and Genomic Science, University of Birmingham, Birmingham, UK; 9 Hubrecht institute-KNAW and University Medical Center Utrecht, Utrecht, The Netherlands; 10 Amsterdam UMC location University of Amsterdam, Department of Surgery, Cancer Center Amsterdam, Meibergdreef 9, Amsterdam, The Netherlands; 11 Amsterdam UMC location University of Amsterdam, Department of Pathology, Cancer Center Amsterdam, Meibergdreef 9, Amsterdam, The Netherlands; 12 Department of Surgical Oncology, UMC Utrecht Cancer Center, University Medical Center Utrecht, Utrecht, The Netherlands; 13 Amsterdam UMC location University of Amsterdam, Department of Oncogenomics, Meibergdreef 9, Amsterdam, The Netherlands; 14 Department of Epidemiology, Julius Center for Health Sciences and Primary Care, University Medical Center, Utrecht University, Utrecht, The Netherlands; 15 Department of Surgery, Catharina Hospital, Eindhoven, the Netherlands; GROW - School for Oncology and Developmental Biology, Maastricht University, Maastricht, Netherlands; 16 Amsterdam UMC location University of Amsterdam, Department of Medical Oncology, Cancer Center Amsterdam, Meibergdreef 9, Amsterdam, The Netherlands

**Keywords:** Colon cancer, Metastasis, Bioinformatics, Cancer genomics

## Abstract

A significant proportion of colorectal cancer (CRC) patients develop peritoneal metastases (PM) in the course of their disease. PMs are associated with a poor quality of life, significant morbidity and dismal disease outcome. To improve care for this patient group, a better understanding of the molecular characteristics of CRC-PM is required. Here we present a comprehensive molecular characterization of a cohort of 52 patients. This reveals that CRC-PM represent a distinct CRC molecular subtype, CMS4, but can be further divided in three separate categories, each presenting with unique features. We uncover that the CMS4-associated structural protein Moesin plays a key role in peritoneal dissemination. Finally, we define specific evolutionary features of CRC-PM which indicate that polyclonal metastatic seeding underlies these lesions. Together our results suggest that CRC-PM should be perceived as a distinct disease entity.

## Abstract (web summary)

Colorectal cancer can lead to the development of peritoneal metastases, which are associated with worse disease outcome. Here, the authors characterize peritoneal metastases from 52 patients using RNA-seq and mutational sequencing and show a distinct molecular subtype.

## Introduction

Colorectal cancer (CRC) is the third most common cancer type worldwide, with an incidence of more than 1.9 million annually (2020)^1^. CRC is the second cause of cancer-related death, of which most are caused by disseminated disease. In addition to hepatic and pulmonary metastasis, peritoneal seeding is one of the most common form of metastasis in CRC (up to 10% of all CRC patients)^2^. Whereas around 5% of CRC patients are diagnosed with synchronous peritoneal metastases (PM), a similar proportion develops metachronous peritoneal metastases at later stages of the disease^3–9^. Importantly, peritoneal metastases are notoriously difficult to diagnose by routine imaging, so this is likely an underestimation of the true incidence^10^.

Peritoneal metastases are associated with significant morbidity, including pain, impaired bowel function, and intra-abdominal fluid accumulation (ascites). Furthermore, patients presenting with peritoneal metastases have a worse overall survival compared to CRC patients with dissemination to other organs^11^. This can be partially explained by the fact that PM is often associated with advanced metastatic disease involving multiple organs. In addition, systemic therapy shows impaired efficacy for peritoneal metastases as compared to other disease locations^11^. Although peritoneal-specific treatment methods, such as extensive cytoreductive surgery (CRS) followed by hyperthermic intraperitoneal chemotherapy (HIPEC) can result in a 5-year survival rate up to 30–50% in patients with limited peritoneal disease, recurrent disease is very common (50–90%)^12,13^. Given the paucity of effective treatment options for CRC-PM patients, a better understanding of the biology of peritoneal metastatic disease is urgently required to identify risk factors for the development of peritoneal metastases, and to identify therapeutic targets for this subgroup of CRC patients.

In contrast to systemic dissemination resulting in liver or lung metastasis, where tumor cells need to enter the blood circulation, direct seeding into the peritoneal cavity is thought to be the most important pathway for peritoneal metastases^14^. However, other routes of dissemination, e.g., by spread through lymphatics situated in the peritoneum may also be of importance. Reported risk factors for peritoneal seeding include tumor cell spillage during surgery of the primary CRC, locally advanced tumor stage (T4), lymph node metastases, right sided, mucinous or signet cell cancers, or aberrations in KRAS/BRAF signaling^5,7,15–21^. Successful peritoneal dissemination seems to require distinct biological properties, such as the ability to survive as free-floating cells, the capacity to adhere to and invade other tissues, to create a suitable microenvironment including neo-angiogenesis, as well as to evade the immune system during this process^14^. Given that only a proportion of CRC patients develops peritoneal metastases, there might be a predefined subgroup of high-risk patients, based on the molecular characteristics of the tumor. Previously, we contributed to the development of the CRC Consensus Molecular Subtypes (CMS) classification system, which defines CRCs based on their transcription profiles^22^. The resulting four subgroups (CMS1-4) are characterized by distinct molecular and biological features as well as clinical outcome. In short, CMS1 is associated with immune activation and microsatellite instable (MSI) tumors, the canonical CMS2 is characterized by epithelial cancers with high WNT and MYC activation, whereas CMS3 mainly comprises epithelial tumors displaying metabolic alterations. The mesenchymal subtype (CMS4) is characterized by strong TGF-β activation, immune suppression, and stromal invasion. Clinically, CMS4 is associated with worse overall and relapse-free survival and resistance to commonly used chemotherapeutic agents^22,23^.

CMS classification of liver metastases revealed that the majority of the lesions is CMS2 (>60%), the rest CMS4, and CMS1 and 3 were virtually absent^24^. In contrast, quantitative PCR analysis of peritoneal metastasis patient material revealed a high presence of mesenchymal tumors (60% of primary CRCs and 75% of peritoneal metastases)^25^. Nevertheless, an extensive transcriptomic and genomic analysis of CRC-associated peritoneal metastases in order to stratify different patient groups is currently lacking.

We present here an in-depth molecular characterization of a cohort of 52 CRC-PM patients and demonstrate that despite CRC-PM being a heterogeneous disease, CMS4 is the predominant subtype. We demonstrate that, in contrast to liver metastases, peritoneal metastases closely recapitulate the primary cancer. Furthermore, we define expression of the structural protein Moesin as the CMS4-specific determinant that allows for spread to the peritoneum of these CRCs. Finally, we identify 3 subgroups within CMS4 peritoneal metastasis patients, with distinct molecular and clinical features that capture the clinical heterogeneity in CRC-PM and could direct future therapy development.

## Results

### CRC-associated peritoneal metastases are enriched in CMS4 cancers

**Fig. 1:**
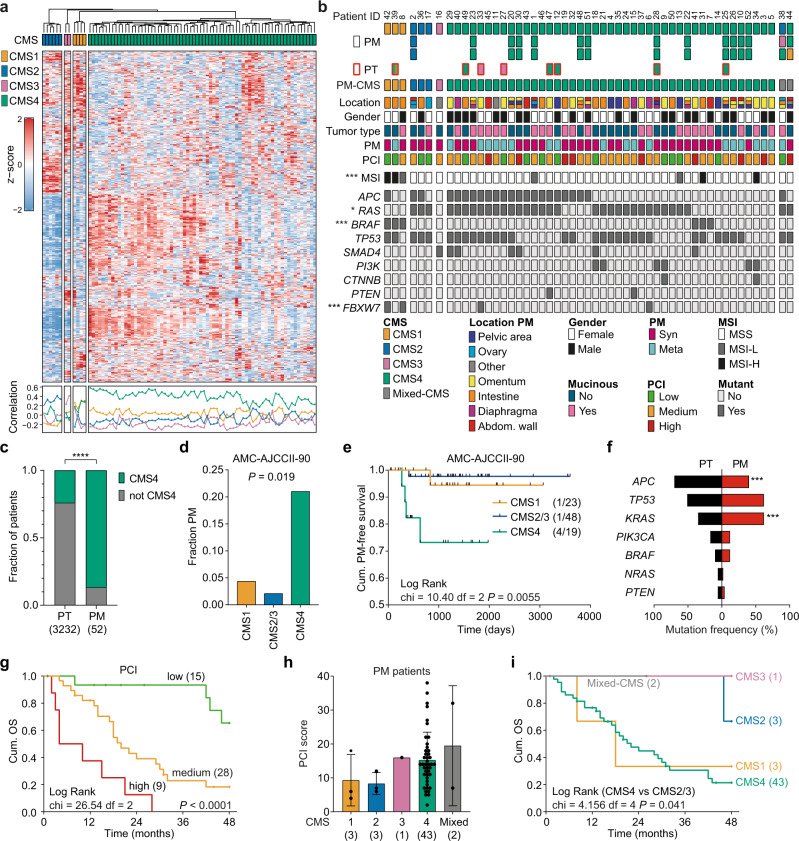
Molecular characterization of peritoneal metastasis tissue. **a** CMS classification of 82 fresh frozen peritoneal metastasis samples (CMSclassifier - Single Sample Prediction (SSP)). Samples are clustered by nearest CMS classification, indicated by the colors on top. Heatmap showing the expression of the top 500 most differentially expressed genes within the peritoneal metastasis group. Correlation of samples with the CMS subtypes is depicted below the heatmap, colors of lines correspond with CMS subtype. **b** Schematic overview of 90 CRC-PM patient samples (52 patients). Squares represent individual tissue samples, colors indicate predicted CMS subtype. Metastasis samples (PM, *n* = 82, black outline) and primary tumor tissue (PT, *n* = 8, red outline). Location of PM, gender, primary tumor type, synchronous or metachronous development of peritoneal metastasis and PCI scores are depicted. MSI status and drivers gene mutations were derived from 1 sample per patient. Asterisks indicate significant differences between CMS subtypes (two-sided Chi-square, MSI ***P = 0.0071; RAS *P = 0.043; BRAF ***P = 5.89E-05; FBXW7 ***P = 0.0034). **c** Frequency of CMS4 tumors amongst primary CRC (Guinney, *n* = 3232) and peritoneal metastasis (*n* = 52) (two-sided Chi-square, *P* < 0.0001). **d**, **e** Incidence of peritoneal metastasis (PM) (**d**) and PM free survival (**e**) of stage II CRC patients (*n* = 90, AMC-AJCCII-90 cohort) stratified to CMS1, CMS2/3 and CMS4 (two-sided Fisher’s exact test, (**d**) and Log-rank (Mantel-Cox) test (**e**)). **f** Mutation frequencies of common driver genes in primary CRC tumors (PT, black bars) and peritoneal metastasis (PM, red bars, two-sided Fisher’s exact test, APC ***P = 0.0004; KRAS ***P = 0.0002). **g** Overall 4-year survival of peritoneal metastasis patients (*n* = 52), stratified to PCI score (low: <10, medium: 10–20, high: >20; Log Rank test, *P* < 0.0001). **h** Bar plots showing PCI score (mean and standard deviation) in relation to patient PM-CMS classification. **i** Overall 4-year survival of peritoneal metastasis patients (*n* = 52), stratified to patient PM-CMS classification (Log Rank test, *P* = 0.289). Source data are provided as a Source data file.

We collected 82 fresh frozen peritoneal metastasis samples and 8 matching primary CRCs of 52 CRC patients that were treated at the Amsterdam UMC. Clinical characteristics of the cohort are described in Supplementary Data 1. CMS was determined of either a single (*n* = 37) or multiple peritoneal lesions (*n* = 15, 3 samples/patient), based on the RNA transcription profiles (Fig. 1a). Most peritoneal metastases classified as CMS4 (85.4%, 70/82 samples), although transcriptional heterogeneity between CMS4 samples was observed (Fig. 1a). Concordance between multiple samples from the same patient was high, 13/15 patients with multiple samples showed the same classification in all lesions (Fig. 1b). We classified 82.6% (43/52) of the peritoneal metastasis patients as CMS4, whereas CMS1 (3/52, 5.8%), CMS2 (3/52, 5.8%), CMS3 (1/52, 1.9%), or mixed CMS (2/52, 3.8%) were much less frequent (Fig. 1b). Compared to CMS classification of a large series of stage I-IV primary CRC samples (*n* = 3232 patients)^22^, CMS4 was highly enriched in the metastasis samples of the CRC-PM patient group (*n* = 52 patients) (*P* < 0.0001, Fig. 1c) as well as in corresponding primary tumor tissue (6/8 patients) (Fig. 1b). These results suggest that CMS4 cancers frequently present with peritoneal disease, which is supported by a significantly higher incidence of peritoneal metastasis formation in CMS4 patients in a cohort of stage II CRC primary tumor patients (*n* = 90, AMC-AJCCII-90^26^) (Fig. 1d, e). CMS classification of both primary CRC and matching PM tissue of an independent cohort^27^, further validated these findings (Supplementary Fig. 1).

### Mutation analysis in peritoneal metastases

Mutation analysis using an actionable mutation panel was performed for each patient (Supplementary Data 2). CMS1 classification was strongly correlated with MSI and the *BRAF-V600E* mutation (Fig. 1b and Supplementary Data 3). The remaining MSI samples (*n* = 4) were all CMS4 and originated from mucinous tumors (Fig. 1b). *KRAS* and *TP53* mutations were found in all CMS2 patients (*n* = 3) (Fig. 1b). The only CMS3 patient was characterized by a *SMAD4* mutation, but no other oncogenic mutations were detected in this sample. Compared to the aggregate cohort representing all stages of CRC used in Guinney et al.^22^, we detected less *APC* mutations in the peritoneal metastasis cohort (44.2% vs 70.0% mutated, *P* = 0.0004), although this might be attributed to the targeted sequencing method. Whereas *BRAF* mutations occurred at a rate similar to what has been reported previously in CRC patients (11.5 vs 10.0% mutated), we found slightly less mutations in *NRAS* (1.9% vs 6% mutated), and a markedly increased *KRAS* mutation frequency in the peritoneal metastasis cohort (61.5% vs 35.0% mutated, *P* = 0.0002) (Fig. 1f and Supplementary Data 4). This is also considerably higher than recently reported mutation frequencies of *KRAS* in metastatic CRC (*KRAS* 35.9%, *BRAF* 7.1%, and *NRAS* 4.1%)^28^. In line with these observations, we detected enrichment of *KRAS*/*BRAF* mutations and increased KRAS signaling activity in primary stage II tumors (AMC-AJCCII-90) that will eventually recur as peritoneal metastatic disease (Supplementary Fig. 2a, b). These findings suggest that KRAS activation represents a predictive factor for peritoneal dissemination.

### CMS subtypes in CRC-PM are associated with clinicopathological features and survival

Metastatic lesions demonstrated clear morphological differences between CMS subtypes, with organized glandular structures in CMS2 and CMS3 samples, increased number of stromal cells in CMS4 lesions (Supplementary Fig. 2c), as well as reduced tumor cell content of CMS4 samples (Supplementary Fig. 2d). Location of the primary tumor and CMS classification of the peritoneal metastasis were not correlated, although CMS1 peritoneal lesions appeared to originate more often from right-sided tumors, in line with the enrichment of MSI cancers in this subtype (Supplementary Fig. 2e). While most peritoneal lesions were located at the peritoneum covering the intestine or the omentum, the majority of CMS2-classified peritoneal metastases were derived from the ovaries suggesting CMS-subtype-specific tropism in the seeding process (Supplementary Fig. 2f).

A significant enrichment of mucinous primary tumors was present in the peritoneal metastasis cohort, compared to the TCGA primary colon cancer (COAD) dataset^29^ (48.1% vs 13.2%, *P* < 0.0001; Supplementary Fig. 2g). The mucinous adenocarcinoma type has previously been associated with CMS4, MSI, mutations in the RAS-MAPK pathway, worse overall prognosis and increased frequency of peritoneal metastasis^21,30,31^. Although mucinous adenocarcinoma was not associated with MSI or *RAS* mutations in our peritoneal metastasis cohort (*P* = 0.241 and *P* = 0.774), it was correlated with the PCI (Peritoneal Cancer Index^32^) score, which is the combined score of the peritoneal tumor load and size (*P* = 0.031, Supplementary Fig. 2h). PCI score is strongly associated with overall survival (OS) (Fig. 1g), and recurrence-free survival (RFS) (Supplementary Fig. 2i). In contrast, no differences in OS or RFS between patients with meta- or synchronous peritoneal metastases were found (Supplementary Fig. 2j). Mutations in *RAS* or *BRAF* did not affect OS, however, we did find that the few patients with *RAS*/*BRAF* wild-type cancers showed a worse RFS (*P* = 0.006) and a significantly increased tumor load (PCI score, *P* = 0.031) (Supplementary Fig. 2k, l).

Although we did not find a statistically significant correlation between PCI score and CMS classification due to low numbers of non-CMS4 patients, most patients with high PCI scores were classified as CMS4 (Fig. 1h). In accordance, CMS4 patients display a worse OS compared to CMS2, CMS3 or mixed-CMS patients (Fig. 1i, CMS4 vs CMS2/3: *P* = 0.041). As has been reported before for metastatic CMS1 CRC^33–35^, also CMS1 patients in the peritoneal metastasis cohort showed a poor OS (Fig. 1i).

### CMS4-specific Moesin (MSN) expression is functionally implicated in peritoneal metastasis formation

**Fig. 2:**
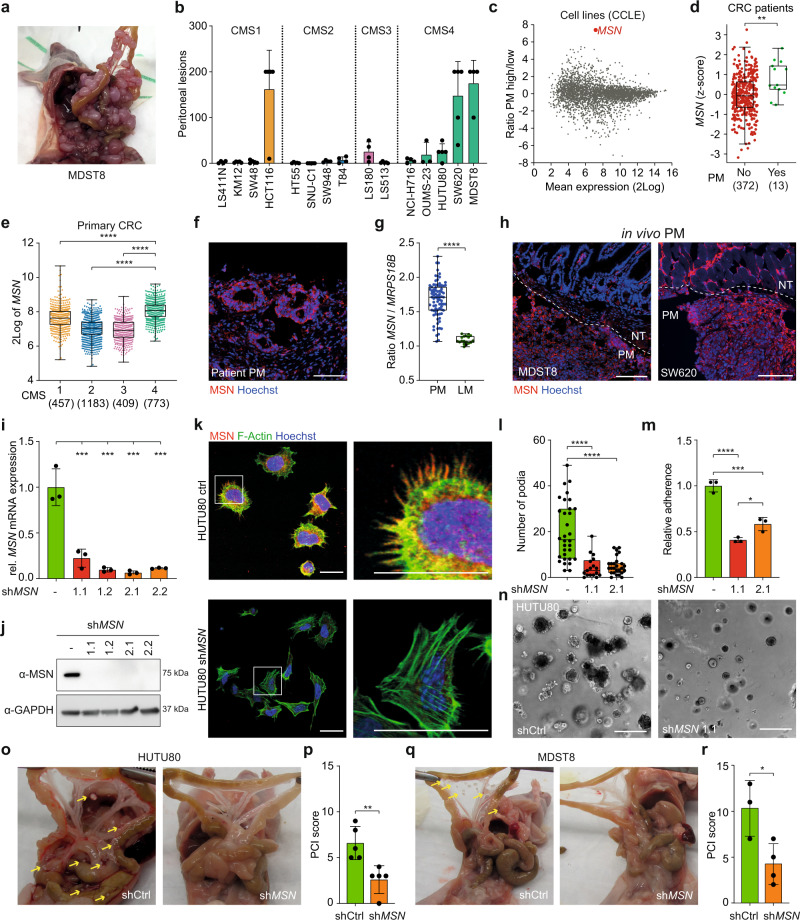
Subtype-specific MSN expression is related to peritoneal metastasis. **a** Peritoneal outgrowth of MDST8 cells, 10 weeks post i.p. injection. **b** CMS specific peritoneal outgrowth of CRC cell lines, 10 weeks post injection (mean ± S.D., *n* = 5 animals for all cell lines, except LS411N, SW948, T84, NCI-H716, MDST8 (*n* = 4 animals) and OUMS23 (*n* = 3 animals)). **c** MA-plot showing all differentially expressed genes (CCLE cell line panel) between PM high (>10 lesions/animal: HCT116, LS180, OUMS-23, HUTU-80, MDST8, SW620) vs low (≤10 lesions/animal: LS411, KM12, SW48, HT55, LS513, SW948, T84, SNU-C1, NCI-H716). **d** *MSN* expression (z-score) in primary CRCs of patients with and without PM (AMC-AJCCII-90 combined with MATCH cohort, *n* = 385, two-sided Mann–Whitney U test, *P* = 0.0034). **e** *MSN* expression (2Log) in primary CRCs^22^, stratified to CMS (one-way ANOVA with Tukey’s multiple comparisons test). **f** MSN expression in patient-derived fixed frozen peritoneal tumor material, MSN (red), nuclear staining (Hoechst, blue). Scale bars, 100 µm. **g** Ratio of gene expression (2Log) of *MSN* and housekeeping gene *MRPS18B*, in peritoneal metastases (PM, *n* = 82) and liver metastases (LM, *n* = 18, Kim et al.^69^) (unpaired, two-sided t-test, *P* < 0.00001). **h** MSN expression in MDST8 and SW620 in vivo peritoneal lesions, MSN (red), nuclear staining (Hoechst, blue), white dotted line indicates border between normal tissue (NT) and peritoneal metastasis (PM). Scale bars, 100 µm. **i**, **j** Two different sh-RNAs targeting *MSN* were lentivirally transduced into HUTU80 cells, single cell clones were established and MSN expression was analyzed on both mRNA (**i**) and protein level (**j**). *GAPDH* was used as a housekeeping gene to normalize mRNA expression (**i**, one-way ANOVA with Tukey’s multiple comparisons test *P* = 0.009, 0.0003, 0.0002, and 0.0003, respectively). **k**, **l** Confocal images (**k**) of either control or *MSN* knockdown HUTU80 cells, 24 h after seeding, MSN (red), F-Actin (green) and nuclei (Hoechst, blue). Right images are magnifications of white boxes in left images. Scale bars, 25 µm. **l** Number of filopodia per cell (*n* = 32, 17, or 26 cells/condition, for respectively control, sh*MSN*1.1 and sh*MSN*2.1, one-way ANOVA with Tukey’s multiple comparisons test, ****P < 0.0001). **m** Relative adherence of HUTU80 cells is decreased upon *MSN* knockdown (one-way ANOVA with Tukey’s multiple comparisons test, ****P < 0.0001, ***P = 0.0003, *P = 0.0265). **n** 3D matrigel outgrowth of *MSN* knockdown HUTU80 is impaired. Scale bars, 50 µm. **o–r** In vivo peritoneal tumor outgrowth is impaired by *MSN* knockdown for HUTU80 (**o**, **p**) and MDST8 (**q**, **r**). Representative images of tumor burden (**o**, **q**) and PCI score (**p**, **r**) of mice injected with respectively HUTU80 or MDST8 control or *MSN* knockdown cells, yellow arrows indicate lesions (**p**, *n* = 5 animals, **r**, *n* = 3 (control) or 4 (sh*MSN*) animals, two-sided *t*-test, *P* = 0.0054 (**p**); *P* = 0.0274 (**r**)). **b**, **i**, **m**, **p**, **r** Bar plots represent mean and standard deviation. **d**, **e**, **g**, **l** Boxplots indicate median, first and third quartiles (Q1 and Q3), whiskers extend to the furthest values. Source data are provided as a Source data file.

The high prevalence of CMS4, mucinous tumors, and activating mutations in the RAS-BRAF axis suggests selection for a specific tumor type during the peritoneal dissemination process. This was further supported using an in vivo peritoneal metastasis model that we recently developed^36^. Cell lines classified as CMS4 were enriched amongst the cell lines displaying enhanced ability to form peritoneal metastases in this assay (Fig. 2a, b), supporting the notion that the ability to seed to the peritoneal lining is an epithelial intrinsic, CMS4-related feature. To identify putative determinants of peritoneal spread, we performed differential gene expression analysis comparing CRC cell lines able to develop high numbers of peritoneal lesions in vivo, and cell lines that are not (Fig. 2c, Supplementary Data 5 and Supplementary Fig. 3a, b). This analysis revealed *MSN* (Moesin; membrane-organizing extension spike protein) as most differentially expressed between cell lines that do or do not form peritoneal metastases (Fig. 2c). Together with *EZR* (Ezrin) and *RDX* (Radixin), *MSN* comprises the ERM family, which members are reported to function as cross-linkers between transmembrane proteins, i.e., CD44, EGFR or other receptor tyrosine kinases, and the actin cytoskeleton, thereby regulating processes such as adhesion and cell migration, and all members have been implicated in cancer progression^37–42^.

*MSN* was significantly higher expressed in primary tumors of peritoneal metastasis patients (Fig. 2d) and CMS4 primary CRCs (Fig. 2e), indicating that *MSN* expression is not increased in peritoneal lesions but already present in the primary cancers from which the peritoneal metastases derive. Staining of patient peritoneal metastasis tissue confirmed the abundance of Moesin-positive tumor cells (Fig. 2f). Moreover, *MSN* expression was also strongly increased in peritoneal metastases compared to liver metastases (Fig. 2g).

MSN protein expression was most abundant at tumor borders in the in vivo peritoneal tumors, especially where tumor cells interact with the surrounding tissue (Fig. 2h). Similarly, in vitro MSN expression was most prominent in cells at or outside the border of cell colonies (Supplementary Fig. 3c). Functionally, MSN is involved in the adherence of cells to extracellular matrix components, as knockdown of *MSN* resulted in reduced filopodia formation after seeding, decreased adherence to collagen-coated surfaces and reduced 3D growth (Fig. 2i–n and Supplementary Fig. 3d–f). In contrast, ectopic expression of *MSN* in CMS2 cell lines resulted in enhanced in vitro filopodia formation (Supplementary Fig. 3g, h), again emphasizing the role of MSN during cell attachment. *MSN* knockdown resulted in reduced in vivo peritoneal metastasis formation (Fig. 2o–r), whereas this did not affect in vitro outgrowth or subcutaneous tumor growth rate in vivo (Supplementary Fig. 3i–k). Altogether, this data indicates a requirement for CMS4-related *MSN* expression during peritoneal dissemination.

### Distinct subgroups can be identified within CMS4 peritoneal metastases

**Fig. 3:**
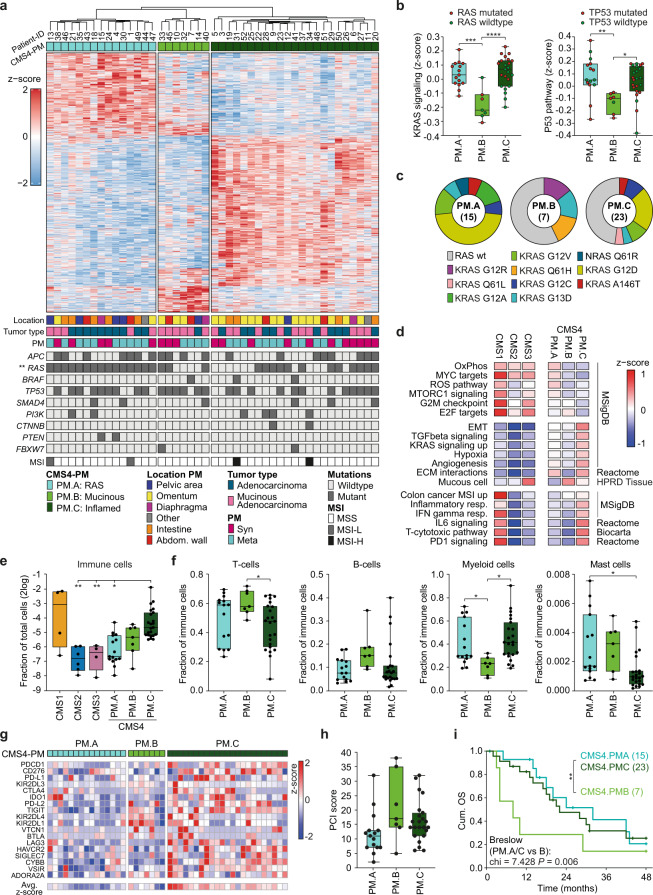
Clustering CMS4 peritoneal metastases into 3 distinct subgroups. **a** Heatmap depicting the 1355 most differential expressed genes between the 3 CMS4-PM subgroups (1 PM sample per patient, *n* = 45), clustered by CMS4-PM subgroup. Location of lesions, primary tumor type, development of PM (syn- or metachronous), mutational status of CRC driver genes and MSI are indicated below (two-sided Fisher’s exact test). **b** Z-scores of KRAS (left) and TP53 (right) activation gene signatures in the CMS4-PM subgroups. Red and green dots indicate respectively mutated or wild-type genes (one-way ANOVA with Tukey’s multiple comparisons test, ***P = 0.0001, ****P < 0.0001, **P = 0.0049, *P = 0.0232). **c** Distribution of *RAS* mutations (*KRAS* and *NRAS*) over the 3 CMS4-PM subtypes. **d** Gene set enrichment analysis depicting differentially expressed (*P* < 0.05, two-sided t-test) signatures between the PM subgroups. Colors indicate relative signature expression (z-score). **e** Deconvolution of bulk RNA sequencing data was used to determine the fraction of immune cells per sample (Welch ANOVA with Dunnett’s multiple comparisons test, *P* = 0.0036, *P* = 0.0048, and *P* = 0.0166). **f** Relative distribution of immune cell types over total immune cell compartment (one-way ANOVA with Tukey’s multiple comparisons test, T cells *P = 0.049; Myeloid cells *P = 0.0476 and *P* = 0.0112; Mast cells *P = 0.0142). **g** Heatmap depicting relative expression of 18 immune checkpoint-associated genes within CMS4-PM subgroups. **h** Boxplots showing PCI scores within the 3 CMS4-PM subgroups (one-way Anova, *P* = 0.058). **i** Cumulative overall survival (OS) of patients with CMS4 classified peritoneal lesions, stratified to the 3 CMS4 subgroups. CMS4-PM.B has a significantly worse survival when compared to the other two combined (Breslow, *P* = 0.006). **b**, **e**, **f**, **h** *n* = 15 (CMS4-PM.A), *n* = 7 (CMS4-PM.B) or *n* = 23 (CMS4-PM.C) biologically independent samples. **a**, **b**, **e**, **f**, **i** Asterisks indicate level of significance: *P < 0.05; **P < 0.01; ***P < 0.001; ****P < 0.0001. **b**, **e**, **f**, **h** Boxplots indicate median, first and third quartiles (Q1 and Q3), whiskers extend to the furthest values. Source data are provided as a Source data file.

Patients with peritoneal metastases present with marked clinical variation in progression rate and symptoms. Given the extensive heterogeneity in transcription profiles of CMS4 peritoneal samples (Fig. 1a), we assessed the possibility of the presence of multiple CMS4 sub-clusters. We found an optimal number of 3 different CMS4 clusters (CMS4-PM.A, B, and C) with distinct molecular and clinical features (Fig. 3a and Supplementary Fig. 4a, b). Hopkins statistics for clustering tendency of the dataset confirmed the validity of sub-clustering the CMS4-PM samples, using the original CMS cohort as a positive control (Supplementary Fig. 4c)^22^. Compared to CMS4-PM.A, we observed a preference for the omentum as the site of metastasis in the CMS4-PM.C subgroup, whereas no bias towards tumor cell content or location of primary tumor was observed in the clustering (Fig. 3a, Supplementary Fig. 4d–f and Supplementary Data 5). On the other hand, we did find enrichment for lesions originating from mucinous primary tumors in CMS4-PM.B, which was also reflected in the expression of genes coding for secreted mucins (*MUC2, MUC5AC, MUC5B*, and *MUC6*) in these samples (Fig. 3a, Supplementary Fig. 4g, h and Supplementary Data 6). Mutation analysis revealed a striking enrichment of *KRAS* mutant samples in the CMS4-PM.A cluster (15/15 samples contain a *KRAS* mutation) and a relatively high frequency of *TP53* mutations in the CMS4-PM.B cluster (Fig. 3a, b, Supplementary Fig. 4i, j and Supplementary Data 6). In support, a clearly decreased pathway activity for KRAS and TP53 was observed in the CMS4-PM.B cluster, whereas the specific *KRAS* mutations (G12R, G13D, and Q61H) found in this cluster had a low frequency in the total cohort and did not result in increased KRAS signaling scores, suggesting a different, less KRAS dependent biology in this group (Fig. 3b, c).

Further gene set enrichment analysis revealed high expression of the oxidative phosphorylation, Myc, and reactive oxygen species (ROS) pathways in CMS4-PM.A (*RAS* mutant subtype). Enrichment of DNA replication processes and E2F targets, reduced KRAS and TP53 signaling scores, and enrichment for mucous cell type signature were found for the mucinous subtype (CMS4-PM.B) (Fig. 3d). CMS4-PM.C exhibited the highest expression levels of epithelial–mesenchymal transition (EMT)-, TGF-β-, KRAS-, and immune-related pathways (inflamed subtype) (Fig. 3d). Deconvolution of bulk RNA sequencing revealed that next to CMS1 lesions, samples from the CMS4-PM.C cluster have the highest infiltration of immune and stromal cells (Fig. 3e and Supplementary Fig. 5a–d). Further deconvolution of the immune cell compartment demonstrated that also the composition of the immune cell population was different between the CMS4 subgroups, with a reduced relative abundance of myeloid cells in CMS4-PM.B (Fig. 3f and Supplementary Fig. 5e). Gene expression of inhibitory molecules including *PD-1*, *PD-L1*, *CTLA4*, *TIM-3*, or *TIGIT* was upregulated in CMS4-PM.C (inflamed subtype) samples, suggesting an immunosuppressive microenvironment in these tumors (Fig. 3g).

Clinically, patients with CMS4-PM.B lesions presented with higher PCI scores (Fig. 3h and Supplementary Data 6), and a worse OS compared to the other 2 clusters combined (*P* = 0.006) (Fig. 3i).

### Peritoneal metastases recapitulate primary CRC phenotype and composition

**Fig. 4:**
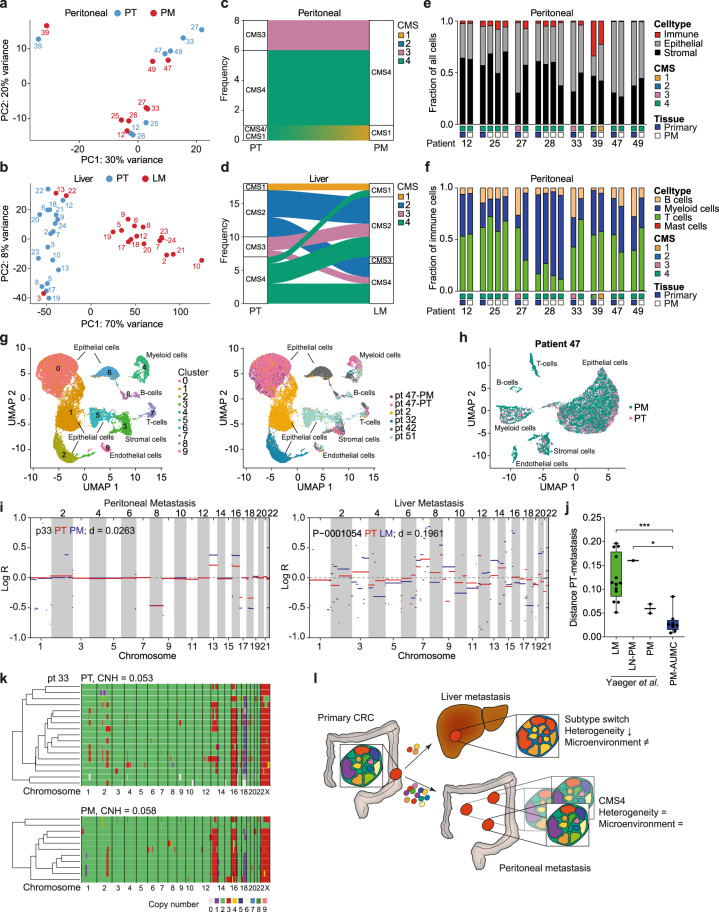
Primary tumor and peritoneal metastasis have highly similar profiles. **a**, **b** PCA plots of matching primary CRC (PT, blue) and peritoneal metastasis (PM, red, *n* = 8 patients, **a**), or liver metastasis (LM, red, *n* = 18 patients, Kim et al.^69^, **b**). Numbers correspond to patient ID. **c**, **d** Alluvial plot showing CMS classification of matching primary CRC (PT) and peritoneal metastasis samples (PM, *n* = 8 patients, **c**) or liver metastasis (LM, *n* = 18 patients, **d**). **e**, **f** Deconvolution of RNA sequencing data was used to compare total cellular composition (**e**) or immune cell composition (**f**) of matched primary CRC (blue box) and PM (white box) samples, CMS of sample is indicated by colored boxes. **g**, **h** Single-cell transcriptome analysis was performed on 6 CRC samples (1 primary tumor, 5 PM samples). **g** UMAP (Uniform Manifold Approximation and Projection) plot of 26,570 cells from all 6 samples, color-coded by clusters (left panel) or sample ID (right panel). **h** UMAP plot of 11,689 cells from patient 47 (primary tumor (PT) and peritoneal metastasis (PM) sample), color-coded by sample type. **i** CNV profiles of primary CRC (PT, red lines) and matching metastasis sample (PM or LM, blue lines) of a PM (left panel) or a LM (right panel) patient. Distance (*d*) between CNV profiles of primary CRC and metastasis sample are depicted. **j** Boxplots depicting the CNV profile distance (*d*) between primary CRC and metastasis samples (median, first and third quartiles (Q1 and Q3), whiskers extend to the furthest values). LM, liver metastasis (*n* = 13 patients); LN-PM, peritoneal metastasis with lymph node metastasis as first metastatic site (*n* = 1 patient); PM, peritoneal metastasis (*n* = 2 patients, Yaeger et al.); PM-AUMC, peritoneal metastasis AUMC dataset (*n* = 8 patients) (one-way ANOVA with Tukey’s multiple comparisons test, ***P = 0.0003, *P = 0.0296). **k** Heatmaps showing hierarchical clustering of single-cell CNV profiles of primary CRC (upper) and matching PM sample (lower) of patient 33. Each row represents a single cell. Colors indicate ploidy number. Copy number heterogeneity was calculated based on single cell CNV profiles and indicated per sample. **l** Schematic representation of peritoneal and liver dissemination. Source data are provided as a Source data file.

Peritoneal metastases develop at the peritoneal surface following the seeding of CRC cells. In contrast to metastases in visceral organs, e.g., the liver, no pre-existing stromal environment with organ resident fibroblasts, immune cells, and endothelial cells is present. Instead, peritoneal lesions attract various cell types, but it is unclear how this relates to the stromal composition at the primary tumor site. To assess this phenomenon, we matched primary tumor and peritoneal metastasis from the same patient. Transcriptome analysis demonstrated high similarity between matching pairs (Fig. 4a). In contrast, liver metastases do cluster together but not with their matching primary tumor reflecting the vastly different microenvironments (Fig. 4b). In line with this, only in a small number of the peritoneal metastasis samples, a CMS-subtype switch compared to the primary tumor (2/8 pairs, both from CMS3 to CMS4) was observed (Fig. 4c), whereas this was much more common for liver metastases (10/18 pairs) (Fig. 4d). Also histologically, peritoneal metastases recapitulate the primary CRC within the same patient (Supplementary Fig. 6). Deconvolution of transcriptome data confirmed a highly conserved cellular composition of primary CRC and peritoneal metastases, both on the level of epithelial-, stromal- and immune- cells, as for immune cell composition (Fig. 4e, f). Subtype-switched metastasis samples showed an increased stromal fraction (Fig. 4e). We conducted single-nucleus transcriptomics analysis on 6 snap-frozen samples, including 1 matching pair of primary CRC and peritoneal metastasis. Clustering analysis of all 26,570 cells revealed the presence of diverse cell types (Fig. 4g and Supplementary Fig. 7a, b). Whereas epithelial cells of different patients were clustered separately, immune and stromal cell types clustered together by cell type, independent of patient origin (Fig. 4g). Epithelial cells from the matching primary tumor and peritoneal metastasis pair clearly clustered together, suggesting high intra-patient similarity, whereas the patient-dependent separation of epithelial cells demonstrates high level of inter-patient heterogeneity (Figs. 4h and 7c, d). In contrast, single-cell data from liver metastasis and matching primary tumors^43^ revealed distinct clusters of epithelial cells derived from primary or metastasized tumor cells implying clonal selection (Supplementary Fig. 7e). Supporting these findings, unbiased sub-clustering of CMS4 primary CRCs resulted in 3 distinct subgroups that highly resemble the 3 CMS4-PM subgroups we identified (Supplementary Figs. 8a–c and 3c). Vice versa, clustering of the PM CMS4 samples using the most differentially expressed genes of the primary CRC CMS4 subgroups, resulted in strongly overlapping subgroups (concordance of 80%, Supplemental Fig. 8d, e). This further suggests that specifically peritoneal metastases recapitulate the phenotype and cell composition of the primary cancer they derive from.

### Peritoneal metastases display low number of copy number aberrations and heterogeneity

Copy number variation (CNV) analysis indicated a highly similar gain/loss frequency profile as found in general CRCs in the TCGA dataset^29^ for both peritoneal and liver metastasis samples (Supplementary Fig. 9a). However, zooming in on individual CNV profiles, we found some striking differences between peritoneal and liver metastasis samples. First, the number of aberrations in both primary tumor and peritoneal metastasis samples was much lower compared to that of patients presenting with liver metastases (Fig. 4i and Supplementary Fig. 9b–d). Similarly, copy number heterogeneity (CNH)^44^ of primary CRC and metastases in CRC-PM patients was significantly lower and more stable compared to liver metastasis patients (Supplementary Fig. 9e). Direct comparison of metastases and matching primary CRC CNV profiles suggests that, compared to liver metastases, peritoneal lesions showed much more similarity to their parental tumor (Fig. 4j). Single cell CNV analysis (scKaryo-seq) of matching primary CRCs and peritoneal metastases confirmed highly conserved CNV profiles and sustained clonal heterogeneity between primary CRC and peritoneal metastases (Fig. 4k and Supplementary Fig. 10).

Overall, these findings suggest a distinct, CMS4-specific dissemination process to the peritoneum compared to the liver, where peritoneal metastases seem to retain both clonal heterogeneity and transcriptional profile of the primary tumor (Fig. 4l). Conversely, liver metastases appear to undergo a more stringent clonal selection, as reported before^45,46^. Together this results in a highly conserved transcriptional and genomic profile between peritoneal metastases and matching primary CRC, strengthening the notion that CRCs presenting with peritoneal metastatic disease represent a distinct disease entity.

## Discussion

In this study, we performed an extensive molecular characterization of a cohort of CRC-derived peritoneal metastases and found that these lesions are predominantly of the CMS4 subtype and enriched for *KRAS* mutations. Unexpectedly, *RAS*/*BRAF* wild-type patients seemed to perform worse after HIPEC (Supplementary Fig. 2k). However, these observations might be due to a bias in our cohort, as patients with high PCI values and *RAS*/*BRAF* mutations might be unfit to undergo diagnostic laparoscopy and potential HIPEC, or might present with more extensive disseminated disease also involving other organs.

We identified the CMS4-specific factor Moesin (*MSN*) as an important component of the peritoneal dissemination process. Interestingly, *MSN* expression has been reported to be repressed by members of the miR200 family^47,48^, whereas oncogenic *KRAS* activation has been reported to suppress the expression of the miR200 family members^49^, suggesting a direct relation between these tumor features. Moreover, miR200-mediated gene regulation is repressed in the mesenchymal CRC subtype (CMS4) as we demonstrated before^50^.

Within the CMS4 subtype, we identified 3 subgroups of peritoneal metastases, respectively characterized by either the presence of *RAS* mutations (CMS4-PM.A), a mucinous phenotype (CMS4-PM.B) or high immune infiltration (inflamed subgroup, CMS4-PM.C). These characteristics are strongly conserved between primary tumor and peritoneal metastasis, implying the CMS4 subtype to be more heterogeneous than previously acknowledged. Clustering tendency analysis on primary tumors (CMS training dataset^22^) did indeed suggest that within the CRC CMS4 subtype a high degree of heterogeneity exists (Supplementary Fig. 4c).

Next to this, our findings also indicate distinct evolutionary paths amongst different types of distant metastases, such as liver and peritoneal metastases. The specific route of dissemination (e.g., via circulation or intraperitoneal seeding), and microenvironment of the distant site will most likely select for a specific population of cells that is able to establish a metastasis. In the liver, this process is driven by clonal selection and monoclonal/single cell seeding, usually resulting in sub-clonal metastases, whereas in the peritoneum, characteristics of the primary tumor seem to define the outgrowth ability, resulting in mainly CMS4 metastases that are highly representative of the primary tumor. Indeed, high tumor heterogeneity of the primary tumor has been associated with increased risk for liver metastasis^51^. The importance of the route of dissemination in determining clonal evolution of metastases is further supported by the dataset of Yaeger et al.^52^, where one of the patients with peritoneal metastases was reported to have lymph node metastasis as first site of dissemination. Interestingly, for this patient, the difference between primary tumor and metastasis was comparable to that of the difference between liver metastases and their matching primary tumor, whereas the two patients in this dataset with peritoneal metastases as the sole site of dissemination displayed much less differences between metastasis and primary tumor, and are comparable to the peritoneal metastasis patients in our cohort (Fig. 4j and Supplementary Fig. 9e).

The finding that peritoneal metastasis-specific traits are already present in primary CRCs, e.g., CMS4 subtype combined with increased KRAS pathway activation (by *KRAS* mutation or downstream events) could be exploited to identify patients at high risk to develop peritoneal metastasis, and who should receive additional (neo)adjuvant therapeutic approaches or more frequent peritoneal inspections by diagnostic laparoscopy or new imaging modalities. We propose that follow-up research into the inhibition of MSN could result in improved clinical strategies to prevent the establishment of peritoneal metastasis.

The subdivision of CMS4 tumors may have further implications for patient stratification. Immune therapy could potentially serve as an interesting treatment option for the inflamed subgroup (CMS4-PM.C), where inhibition of the apparent immune suppression could reactivate immune responses towards the tumor or metastases. The apparent tropism of CMS4-PM.C lesions for the omentum, an organ known to play a role in peritoneal immunity^53^, further suggests a subtype-specific interaction between the immune system and tumor cells within the abdominal cavity. Although further research should clarify the role of the immune system in the development and eradication of peritoneal metastases, some studies indicated the importance of the presence of specific immune cells in the peritoneum. For example, the ratio of tumor infiltrating CD4+ T cells has been associated with improved survival of peritoneal metastasis patients with low tumor load^54^. Other subgroup-specific vulnerabilities might also offer directions towards more personalized therapies, or to development of predictive markers to support early detection.

## Methods

### Patient cohort

Patient samples were collected according to Dutch research guidelines of the Federation of Dutch Medical Scientific Societies (FDMSS), as described in “Human Tissue and Medical Research: Code of Conduct for Responsible use”. When required, patients provided informed consent for sampling additional tumor tissue for study purposes. Patients did not receive any compensation. In this study, we used tumor samples from 52 patients with peritoneal metastases, collected at the Amsterdam University Medical Center, location VUmc between 2010 and 2018. Eligibility criteria for inclusion were: histologically proven colorectal carcinoma with synchronous or metachronous peritoneal metastasis, age older than 18 years, and fresh frozen tissue available. The detailed clinical and histopathological characteristics of this cohort are described in Supplementary Data 1.

### Tissue sample collection

Tissues were obtained from CRC patients with synchronous or metachronous peritoneal metastases. We collected 172 samples from 52 patients, including 8 primary tumors. Fresh tumor tissue was obtained immediately after surgical excision, prior to HIPEC treatment, and after a macroscopic examination a sample selection of the peritoneal metastasis and for some patients also the primary tumors were frozen down for storage at −80 °C.

### Pathological analysis

All samples were reviewed by a gastrointestinal pathologist (L.K.) for tumor content based on hematoxylin and eosin (H&E)-stained tissue sections. From each tumor, an H&E section was available of the top and bottom part of the isolated sample. From both slides the tumor content was reviewed. All samples with an estimated mean cancer cell content above 30% were included for isolation. Data on tumor grade and histology were extracted from the original pathological reports. H&E sections were scanned and exported using Philips Digital Pathology software.

### RNA and DNA isolation

Frozen tissue samples were cut in 20-µm-thick cryosections with a cryostat up to about 30 mg for each sample. All tissue samples were maintained at −80 °C until RNA extraction. Frozen samples were immersed in RLT buffer (AllPrep DNA/RNA/miRNA Universal Kit, Qiagen) and disrupted and homogenized using TissueLyser LT (Qiagen). Total RNA and DNA were isolated simultaneously from tissue lysates using the AllPrep DNA/RNA/miRNA Universal Kit (Qiagen), following the manufacturer’s instructions. The RNA and DNA concentration was measured using NanoDrop 2000 (Thermo Scientific) and Qubit fluorometer (Thermo Fisher Scientific). The RNA integrity was measured using the Agilent RNA 6000 Nano Kit on an Agilent 2100 Bioanalyzer (Agilent Technologies). Only samples with an RIN (RNA integrity number) >6.7 were subjected to further analysis.

### Bulk RNA sequencing, data processing

Libraries were prepared using Kapa mRNA HyperPrep, sequencing was performed using Illumina HiSeq (Single Read, 50 bp). The quality control of the single-end reads was assessed by FastQC (available online at: http://www.bioinformatics.babraham.ac.uk/projects/fastqc/). RNA-seq transcript quantification was performed by mapping the high-quality reads to the GRCh38 human transcriptome by using the software RSEM^55^ and the STAR aligner^56^. The RSEM output corresponding to the liver metastatic samples were downloaded from the recount2 repository^57^ using the accession code SRP029880. The RSEM outputs containing the estimated counts were imported into R (R Core Team, 2020) and summarized into matrices by the R package tximport (v1.18.0)^58^. Detailed description of the RNA sequencing analysis pipeline is available at: https://github.com/vermeulenlab/peritoneal_metastasis^59^.

### CMS classification

Molecular subtype classification was performed based on gene profile expression as described before^22^. Briefly, raw count matrix of RNA-seq was transformed by variance stabilizing transformation^60^ and gene identifiers were converted from gene Symbol to Entrez gene ids using the R package org.Hs.eg.db (genome-wide annotation for Human. R package version 3.8.2. Carlson, 2019). The processed matrix was used as input for the ‘single-sample predictor’ (SSP) classifier, part of the (‘CMSclassifier’ v1.0.0) R package, setting the option method = “SSP”. Since the SSP classifier does not use predefined probabilities for the subtypes, misclassification due to altered distribution of CMSs in the peritoneal metastasis set is avoided. The heatmap for visualizing the gene expression of the most discriminant genes across the CMS subtypes was built by the software ComplexHeatMap (v2.6.2)^61^. Detailed description of the CMS classification methods is available at: https://github.com/vermeulenlab/peritoneal_metastasis^59^.

### Unsupervised sub-clustering of CMS4 samples

Samples of the CMS4 subtype were unsupervised clustered in order to identify intrinsic groups sharing biological characteristics using the R package ConsensusClusterPlus (v1.54.0)^62^, setting clusterAlg = ”pam” and distance = ”pearson” and using the 500 most variable genes across the samples calculated by the R function median absolute deviation (mad). The optimal number of subgroups was further assessed by the R function silhouette. To evaluate the potential clustering of the CMS4 PM dataset (*n* = 45), we used Hopkins statistic (R package “Clustertend” version 1.5) to calculate the score representing the clustering tendency. We compared the obtained Hopkins score in the CMS4 PM dataset against: (1) the Guinney CRC dataset^22^, randomly selecting 1000 samples that were previously classified as one of the CMS subtypes; (2) the Guinney CRC dataset, selecting only samples that were previously classified as CMS4 subtype; (3) a simulated dataset containing uniform data distribution and therefore with low potential clustering. Median absolute deviation (MAD) was used to select the 1000 most variable genes used as input for the calculation. Detailed description of scripts used for the sub-clustering of CMS4 PM samples is available at: https://github.com/vermeulenlab/peritoneal_metastasis^59^.

### Deconvolution of RNA-seq

To conduct the cell type deconvolution of the bulk RNA-seq samples, we employed the deep learning-based method Scaden (v0.9.4)^63^. In short, the processed single-cell data and metadata of 23 colorectal cancer patients were downloaded from the NCBI Gene Expression Omnibus (GEO), accession code GSE132465, and used as a training dataset. The function ‘scaden train’ was applied to construct the Scaden ensemble model using 5000 training steps and 30,000 samples. Scripts used for deconvolution of bulk RNA-seq are available at https://github.com/vermeulenlab/peritoneal_metastasis^59^.

### Single nuclei RNA sequencing and data processing

Nuclei were prepared for 10x Genomics-based single nuclei RNA sequencing analysis according to a previously published protocol^64^. Briefly, each frozen sample was thawed and macerated in CST buffer for 10 min, filtered (70 micron pluriStrainer), and spun at 500 × *g* for 5 min at 4 °C to pellet nuclei. Nuclei were resuspended in the same buffer without detergent, filtered (10 micron pluriStrainer), and counted using AOPI on a Nexcelom Cellometer. Approximately 10,000 nuclei were loaded immediately into each channel of a 10x Chromium chip (10x Genomics) using 5-prime V2 chemistry according to the manufacturer’s protocol (10x Genomics #CG000330). The resulting cDNA and indexed libraries were checked for quality on an Agilent 4200 TapeStation and then quantified and pooled for sequencing on an Illumina NextSeq 550. Single-cell sequencing data were aligned to the human reference genome (GRCh38) and processed using the CellRanger 3.1.0 software from 10x Genomics to generate unique molecular identifier (UMI) counts. The raw gene expression matrices were imported into R (R Core Team, 2020) and further processed by the Seurat R package version 3.2.2 (filter < 10% of mitochondrial gene expression and >200 unique gene counts (nFeature_RNA) < 4000) and normalized by SCTransform with regression for nFeature_RNA and the percent mitochondria. Cell clusters were visualized using the UMAP algorithm with the first 10 principal components as input. The major cell populations were annotated by comparing the gene markers for each cluster, identified using the Seurat function FindAllMarkers, and canonical marker genes. More details on single nuclei RNA-seq data processing are available at https://github.com/vermeulenlab/peritoneal_metastasis^59^. To compare single cells derived from liver metastasis and paired primary tumors publicly available data was used from Che et al.^43^.

### Mutation analysis

NGS was performed using a custom targeted NGS amplicon panel consisting of the following genes: *APC*, *TP53*, *KRAS*, *NRAS*, *BRAF*, *PIK3CA*, *SMAD4*, *FBWX7*, *CTNNB1*, *POLE*, *POLD1*, *PTEN*, *ACVR2A*, *BMPR2*, *RNF43*, *ZNRF3*, *MLH1*, *MSH2*, *MSH3*, *MSH6*, and *PMS2*. DNA libraries were produced using the custom Ion AmpliSeq Panel (Life Technologies, Bleiswijk, the Netherlands) according to the manufacturer’s instructions. Libraries were barcoded (Ion Xpress Barcodes adapters kit, Life Technologies) and quantified using a Qubit dsDNA HS assay kit (Life Technologies). Tumor DNA libraries were sequenced on a 530v1 chip in the Personal Genome Machine system (Ion Torrent, Life Technologies). Torrent suite software v5.10.1 was used for signal processing, run quality reports, and to generate BAM files. Sequences were analyzed using SeqNext software v4.1.2 (JSI Medical Systems GmbH, Ettenheim, Germany). The target sequencing depth was 1500× per amplicon. For mutation calling a variant allele fraction (VAF) cutoff value of 5% was used.

### Microsatellite instability analysis

MSI status of the samples was determined using the MSI Analysis System, version 1.2 (Promega), according to the manufacturer’s instructions. Samples were considered MSI-high (MSI-H), MSI-low (MSI-L), or MSS when more than two, one, or zero out of five markers (BAT-25, BAT-26, MON0-27, NR-21, and NR-24) were instable, respectively.

### DNA copy number analysis

Genomic DNA was isolated from each sample and measured with Illumina GSA Beadchip (Illumina GSA Arrays “Infinium iSelect 24×1 HTS Custom Beadchip Kit”). GenomeStudio was used with standard settings to obtain copy numbers from the SNP data. Generated LogR values were segmented using circular binary segmentation via the DNAcopy R package (version 1.58.0) and frequency plots were generated using the same package. Shallow sequencing and analysis of matching primary CRC and peritoneal metastasis was performed as described previously^65^. In short, DNA from fresh frozen samples was sheared on a Covaris S2 (Covaris), sample preparation was performed with the TruSeq DNA kit V2 (Illumina). After end repair and 39 adenylation, adapter ligation was performed with 1 mL of adapter. Final sequence library amplification was performed with 8 PCR cycles, using the following program: start with 30 s 98 °C, 8 cycles of 10 s 98 °C, 30 s 60 °C, and 30 s 72 °C, end with 5 min 72 °C. Sequence library yield was assessed with a Bioanalyzer DNA 1000 and/or HS DNA (Agilent Technologies). Libraries were equimolarly pooled with 18–22 barcoded samples and 7 pM molarity loaded per lane of a HiSeq Single End Flowcell (Illumina). This was followed by cluster generation on a cBot (Illumina) and sequencing on a HiSeq 2000 (Illumina) in a single-read 50-cycle run mode (SR50). CNVs from liver metastases and matching primary CRCs were obtained from publicly available data^52^. Distances between primary and metastasis copy number profiles were calculated by taking the mean absolute distance between two segmented log2 profiles divided by the standard deviation after correcting for purity and ploidy differences between the samples. This was done for a range of values for sample purity (0.2, 0.21, .., 1) and mean ploidy (1.5, 1.51, …, 5) for both the primary and metastatic profile, and the distance between both profiles was taken as the minimum of all distances across the searching grid. For patients with multiple primary or metastatic samples, the mean of all pairwise comparisons was taken.

### Single-cell karyotyping

Single-cell karyotype sequencing of matching primary and peritoneal metastasis samples from colorectal cancer (CRC) patients was performed as described previously^66^. In short, nuclei were isolated from lysed fresh frozen tumor tissue, single nuclei were sorted and genomic DNA was fragmented and barcoded. Libraries were prepared as described previously and sequenced on an Illumina Nextseq 500 with 1 × 75-bp single-end sequencing. Sequencing reads were aligned to genome build ‘GRCh38.p10’ with bwa (v0.7.12). Quality control was performed with Aneufinder v1.14 with default parameters.

### Gene expression analysis (R2 platform)

To perform differential gene expression analysis on CRC cell lines we made use of a publicly available dataset^67^ (Sanger, GSE36133). To analyze gene expression in CRC patients, we made use of the following RNA-seq datasets: AMC-AJCCII-90 cohort^26^ (GSE33113), MATCH cohort^68^ (EGAS00001002197), TCGA primary colon cancer (COAD) dataset^29^ (http://gdac.broadinstitute.org/), and the Guinney dataset^22^, including 3232 colorectal cancer patients (available at: Synapse, syn2623706) and GSE50760 for matched CRC and liver metastasis samples^69^. Gene set enrichment analysis was done using the R2: Genomics Analysis and Visualization Platform (http://r2.amc.nl) website.

### Cell culture

Cell lines T84, SW48, HT55, SW948, LS180, HUTU80, SW620, and OUMS-23 were cultured in Dulbecco’s modified Eagle’s medium/F-12 medium with L-glutamine, 15 mM HEPES (Thermo-Fisher Scientific) supplemented with 10% v/v fetal bovine serum (Life Technologies), penicillin and streptomycin. Cell lines HCT116, KM12, LS411N, SNU-C1, LS513, MDST8, and NCI-H716 were cultured in RPMI 1640 with L-glutamine, 25 mM HEPES (Thermo-Fisher Scientific) supplemented with 10% v/v fetal bovine serum (Life Technologies), penicillin and streptomycin, 1% D-glucose solution plus (Sigma-Aldrich) and 100 μM sodium pyruvate (Life Technologies). All cell lines were obtained as a kind gift from the Sanger Institute (Cambridge, UK) and authenticated by STR Genotyping and regularly tested for mycoplasma infection. Lentiviral short hairpin RNA constructs against MSN were obtained from Sigma-Aldrich. Lentiviral overexpression of MSN was obtained by PCR cloning of *MSN* cDNA, derived from pHJ320-MSN (Addgene plasmid # 20671) into the LeGo-iCer2 (Addgene plasmid # 27346) vector.

### Cell viability, adherence assays, and 3D growth

For in vitro proliferation assays, 2000 tumor cells/well were seeded in 96-wells plates in 100 µl of medium. At different time points, proliferation was measured using the Cell Titer Blue assay (Promega). Fluorescence signal was measured by fluorescence reader (Biotek). To assess the adherence capacity, 20,000 cells were seeded in 96-wells plates coated with Rat tail collagen I (Corning) in 100 µl of medium. Thirty minutes after seeding, the plate was emptied, washed with PBS, and fresh medium was added. The number of attached cells was measured using the Cell Titer Blue assay (Promega). To assess 3D growth, 1000 cells were plated in a drop of Matrigel (Corning) and the well was filled with complete growth medium. Cells were grown for 7 days and colonies were imaged using the EVOS FL auto imaging system (Life).

### Animal experiments

All animal experiments were approved by the Animal Experimentation Committee at the Academic Medical Center in Amsterdam (AVD118002016493) and conducted in accordance with the national guidelines. Female nude (Hsd:Athymic Nude-*Fox1*^*nu*^) mice (6–12 weeks old) were purchased from Envigo. The mice were housed on a 12 h light-dark cycle at 20–26 °C with 30–70% humidity. Animals were randomly assigned to experimental groups, no blinding was performed during these experiments.

#### Subcutaneous tumor growth

5 × 10^4^ colon cancer cells in medium were mixed at a 1:1 ratio with Matrigel (Corning) and injected subcutaneously into both flanks of female nude (Hsd:Athymic Nude-*Fox1*^*nu*^) mice (6–12 weeks old). Tumor growth was measured twice a week using calipers, using the formula 0.5 × length × width × height. Maximum permitted tumor size of 1000 mm^3^ was not exceeded.

#### Intraperitoneal tumor growth

1 × 10^3^ tumor cells in medium were mixed at a 1:1 ratio with cold Matrigel (Corning) and injected intraperitoneally in female nude (Hsd:Athymic Nude-*Fox1*^*nu*^) mice (6–12 weeks old) as also described previously^36^. Ten weeks after injection, mice were sacrificed and the peritoneum was analyzed for the presence of tumor lesions.

### Immunofluorescent imaging

Immediately after isolation, tumors from in vivo models were fixed using 4%-paraformaldehyde followed by 30% sucrose saturation after which tumors were frozen and 20-µm-thick sections were used for stainings.

For in vitro immunofluorescent imaging, cells growing on glass coverslips were fixed using 4%-paraformaldehyde and permeabilized using 1% Triton X100. Tissue sections or fixed cells were incubated with the indicated primary antibody for 3 h followed by incubation with fluorescently labeled secondary antibodies for 1 h, mounted with ProLong Gold Antifade Mountant (Thermo Fisher Scientific), and imaged using an SP8-X confocal microscope (Leica) and the Leica Application Suite-Advanced Fluorescence software. To detect nuclei, sections were counterstained with Hoechst 33342 (Sigma) (405 nm laser), for F-Actin detection ActinGreen-488 (phalloidin) ready probe (ThermoFisher, 1:1000) (488 nm laser) was used. For HE stainings, frozen tumor sections were stained with hematoxylin and eosin (H&E). The following primary antibodies were used: rabbit anti-MSN (HPA011135, Sigma, 1:100). As secondary antibody goat-anti-rabbit-Alexa546 (A11035, Invitrogen, 1:500) was used.

### cDNA synthesis and quantitative RT-PCR

RNA from cell lines was isolated using the Nucleospin RNA isolation kit (Macherey-Nagel). 1 μg of RNA was used to synthesize cDNA using SuperScript III according to the manufacturer’s protocol (Invitrogen). Quantitative RT-PCR was performed with LC480 SYBR green (Roche) in accordance with the manufacturer’s instructions on a LC480. Used primers were:

GAPDH:

Fw: 5′-CCAGCAAGAGCACAAGAGGAAGAG-3′,

Rev: 5′-CAAGGGGTCTACATGGCAACTGTG-3′.

MSN:

Fw: 5′-TGTAAACCAGAGAGCTGCTGG-3′,

Rev: 5′-GAAGAGCACACATGAGACAGAGAA-3′.

### Western blotting

Protein from cells was extracted using RIPA buffer (Bio-Rad) and protein concentration was determined using BCA kit (Pierce). Equal amounts of protein were separated on precasted polyacrylamide gels (Mini-PROTEAN TGX, Bio-Rad) and transferred to PVDF membranes using the Trans-Blot Turbo Transfer System (Bio-Rad). Membranes were blocked for 1 h using 5% non-fat milk or for phospho-protein detection 5% BSA containing phosphatase inhibitor mix (Thermo Fischer), incubated for 3 h with primary antibodies, washed with TBST, followed by 1 h incubation with horseradish peroxidase-linked secondary antibodies. Chemoluminescence was detected using the ImageQuant Las4000 system (GE Lifesciences) after 1 min incubation with ECL substrate (ECL Western Blotting Substrate, Thermo Scientific Pierce). Primary antibodies used were rabbit anti-MSN (HPA011135, Sigma, 1:100), mouse anti-GAPDH (MAB374/6C5, Merck, 1:1000). Secondary antibodies used were HRP goat-anti-rabbit IgG (4050-05, Southern Biotech, 1:10,000) and HRP goat-anti-mouse IgG (1031-05, Southern Biotech, 1:10,000).

### Statistics

Sample sizes, statistical tests, and definitions of error bars are indicated in the figure legends and calculated using GraphPad Prism 9. All statistical tests were two-sided. *P*-values of <0.05 were considered significant.

Survival analysis was performed using IBM SPSS statistics 26.

### Reporting summary

Further information on research design is available in the Nature Research Reporting Summary linked to this article.

## Supplementary information

Supplementary Info

Reporting Summary

Supplementary Data 1

Supplementary Data 2

Supplementary Data 3

Supplementary Data 4

Supplementary Data 5

Supplementary Data 6

## Data Availability

The sequence libraries generated in this study are publicly available through the National Center for Biotechnology Information (NCBI) Gene Expression Omnibus (GEO) under accession code: GSE183202. Single-cell karyotypes of PM samples are available from EGA, accession number EGAS00001004702, dataset ID EGAD00001006438. Data access for EGAS00001004702 is under controlled access due to the provision of potentially identifiable genotypic or phenotypic data. Access will be provided for academic research use only, and access requests should be directed to Bauke Ylstra (b.ylstra@amsterdamumc.nl). Estimated time before access will be granted is ~2 days. Shallow sequencing data of matching primary CRC and peritoneal metastasis samples are available from SRA, accession number PRJNA841870. Other datasets used in this study are publicly available under accession numbers: GSE36133, GSE33113, EGAS00001002197, GSE50760, GSE132465, GSE178318, SRP029880 [https://www.ncbi.nlm.nih.gov/geo/query/acc.cgi?acc=GSE50760], http://gdac.broadinstitute.org/ for TCGA COAD, and Synapse, syn2623706 [https://www.synapse.org/#!Synapse:syn2623706/wiki/67246] for the Guinney dataset or through the R2: Genomics Analysis and Visualization Platform (http://r2.amc.nl). The remaining data are available within the Article, Supplementary Information or Source data file. Source data are provided with this paper.

## Source data

Source Data

## Code availability

All scripts used for data analysis are available at https://github.com/vermeulenlab/peritoneal_metastasis [10.5281/zenodo.6779129]^59^.
