# Supplementary material for: Molecular characterization of colorectal cancer related peritoneal metastatic disease
Source: Nat Commun. 2022 Aug 4;13:4443. doi: 10.1038/s41467-022-32198-z (PMC9352687; doi:10.1038/s41467-022-32198-z)
Supplement: Supplementary file 1 — Supplementary Info [file 41467_2022_32198_MOESM1_ESM.pdf]

Supplementary Information

Molecular characterization of colorectal cancer related peritoneal metastatic disease

Kristiaan J. Lenos[1,2,], Sander Bach[3], Leandro Ferreira Moreno[1,2], Sanne ten Hoorn[1,2], Nina R. Sluiter[3], Sanne Bootsma[1,2], Felipe A. Vieira Braga[1,2], Lisanne E. Nijman[1,2], Tom van den Bosch[1,2], Daniel M. Miedema[1,2], Erik van Dijk[4], Bauke Ylstra[4], Ruth Kulicke[5], Fred P. Davis[5], Nicolas Stransky[5], Gromoslaw A. Smolen[5], Robert R.J. Coebergh van den Braak[6], Jan N.M. IJzermans[6], John W.M. Martens[7], Sally Hallam[8], Andrew D. Beggs[8], Geert J.P.L. Kops[2,9], Nico Lansu[2,9], Vivian P. Bastiaenen[10], Charlotte E.L. Klaver[10], Maria C. Lecca[1,2], Khalid El Makrini[1,2], Clara C. Elbers[1,2], Mark P.G. Dings[1,2], Carel J.M. van Noesel[11], Onno Kranenburg[12], Jan Paul Medema[1,2], Jan Koster[13], Lianne Koens[11], Cornelis J.A. Punt[14], Pieter J. Tanis[10], Ignace H. de Hingh[15], Maarten F. Bijlsma[1,2], Jurriaan B. Tuynman[3,**] & Louis Vermeulen[1,2,16,*,**]*

1. Amsterdam UMC location University of Amsterdam, Center for Experimental and Molecular Medicine, Laboratory for Experimental Oncology and Radiobiology, Cancer Center Amsterdam, Amsterdam Gastroenterology Endocrinology Metabolism, Meibergdreef 9, Amsterdam, the Netherlands.
2. Oncode Institute, Amsterdam, The Netherlands.
3. Amsterdam UMC location Vrije Universiteit Amsterdam, Department of Surgery, Cancer Center Amsterdam, De Boelelaan 1117, Amsterdam, the Netherlands.
4. Amsterdam UMC location Vrije Universiteit Amsterdam, Department of Pathology, Cancer Center Amsterdam, De Boelelaan 1117, Amsterdam, the Netherlands.
5. Celsius Therapeutics, 399 Binney Street, Cambridge, MA 02139, USA.
6. Department of Surgery, Erasmus MC University Medical Center, Rotterdam, The Netherlands.
7. Department of Medical Oncology, Erasmus MC Cancer Institute, Erasmus MC University Medical Center, Rotterdam, the Netherlands & Cancer Genomics Center, Utrecht, the Netherlands.
8. Surgical Research Laboratory, Institute of Cancer and Genomic Science, University of Birmingham, UK.
9. Hubrecht institute-KNAW and University Medical Center Utrecht, Utrecht, The Netherlands.
10. Amsterdam UMC location University of Amsterdam, Department of Surgery, Cancer Center Amsterdam, Meibergdreef 9, the Netherlands.
11. Amsterdam UMC location University of Amsterdam, Department of Pathology, Cancer Center Amsterdam, Amsterdam, Meibergdreef 9, The Netherlands.
12. Department of Surgical Oncology, UMC Utrecht Cancer Center, University Medical Center Utrecht, Utrecht, the Netherlands.
13. Amsterdam UMC location University of Amsterdam, Department of Oncogenomics, Meibergdreef 9, Amsterdam, The Netherlands.
14. Department of Epidemiology, Julius Center for Health Sciences and Primary Care, University Medical Center, Utrecht University, Utrecht, The Netherlands.
15. Department of Surgery, Catharina Hospital, Eindhoven, the Netherlands; GROW - School for Oncology and Developmental Biology, Maastricht University, Maastricht, Netherlands.
16. Amsterdam UMC location University of Amsterdam, Department of Medical Oncology, Cancer Center Amsterdam, Meibergdreef 9, Amsterdam, The Netherlands.

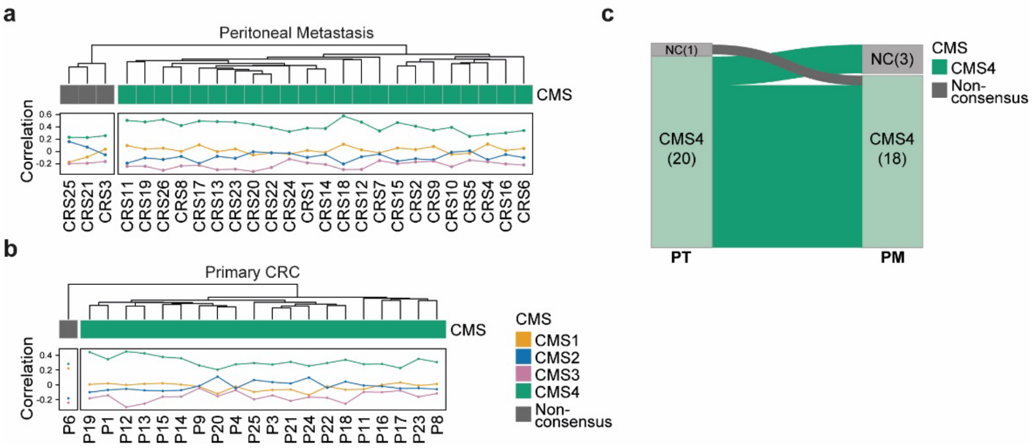

Supplementary Figure 1. CMS classification of an independent cohort of matched CRC and PM samples. a, b Single Sample Predictor (SSP) was applied to 26 PM (**a**) and 21 primary CRC (**b**) samples described in Hallam *et al.*[27] for CMS classification. Predicted CMS is indicated by the colors on top, using a threshold of 0.40. Correlation of samples with each CMS subtype is depicted below, colors of lines correspond with CMS subtype. **c** Alluvial plot showing CMS classification of 21 matching primary CRC (PT) and peritoneal metastasis samples (PM).

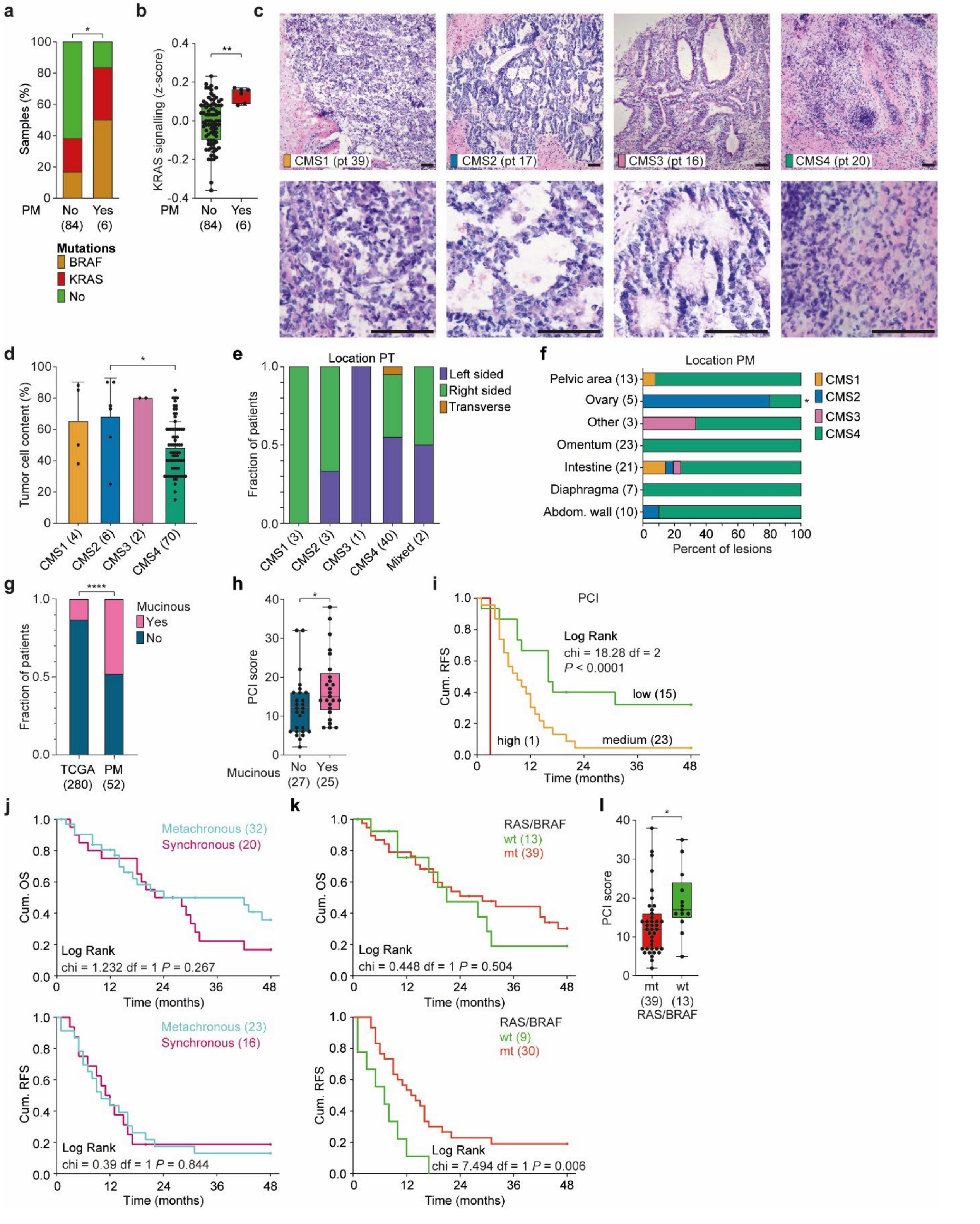

Supplementary Figure 2. Molecular and clinical features of PM cohort. a, **b** Distribution of KRAS and BRAF mutations (**a**) and z-score of KRAS activation (Broad Institute Hallmark) gene signature (**b**) in primary

CRC (AMC-AJCCII-90 cohort), comparing patients that did develop PM (*n* = 6) with those that did not (*n* = 84) (Two-sided Fisher's exact test, *P* = 0.044 (**a**) and unpaired two-sided t-test, *P* = 0.0044 (**b**)). **c** Representative H&E images of peritoneal lesions of the different CMS subtypes. Scalebars, 200 µm (upper panel) and 100 µm (lower panel). **d** Tumor cell content (%) for CMS subtyped PM samples (One-way ANOVA with Tukey's multiple comparisons test, *P* = 0.0498), bar plots represent mean and standard deviation. **e** Localization of primary tumor, stratified to patient PM-CMS subtype (*n* = 49 patients). **f** Location distribution of CMS subtyped peritoneal lesions. Most of the CMS2 samples were found on the ovary (Two-sided Chi-square, *P* = 0.049). **g** Fraction of mucinous adenocarcinomas in patients with primary CRC (TCGA COAD) or PM (Two-sided Fisher's exact test *P* < 0.0001). **h** Boxplots showing PCI score stratified to primary tumor type (adenocarcinoma or mucinous adenocarcinoma, unpaired, two-sided t-test, *n* = 52 patients, *P* = 0.0309). **i** Recurrence free survival (lower panel, *n* = 39 patients) of PM patients stratified to PCI score (Low: PCI <10, medium: PCI: 10-20, high: PCI >20; Logrank test, *P* < 0.0001). **j** OS (upper panel, *n* = 52 patients) and RFS (lower panel, *n* = 39 patients), stratified to timing of PM (syn- or metachronous, Logrank test, respectively *P* = 0.267 and *P* = 0.844). **k** OS (upper panel, *n* = 52 patients) and RFS (lower panel, *n* = 39 patients), stratified to *RAS*/*BRAF* mutational status (wildtype (wt) or mutant (mt), Logrank test, respectively *P* = 0.504 and *P* = 0.006). **l** PCI score for PM patients stratified to *RAS*/*BRAF* mutational status (two-sided unpaired t-test, *P* = 0.0306). **b**, **h**, **l** Box plots represent median, first and third quartiles (Q1 and Q3), whiskers extend to the furthest values. Source data are provided as a Source Data file.

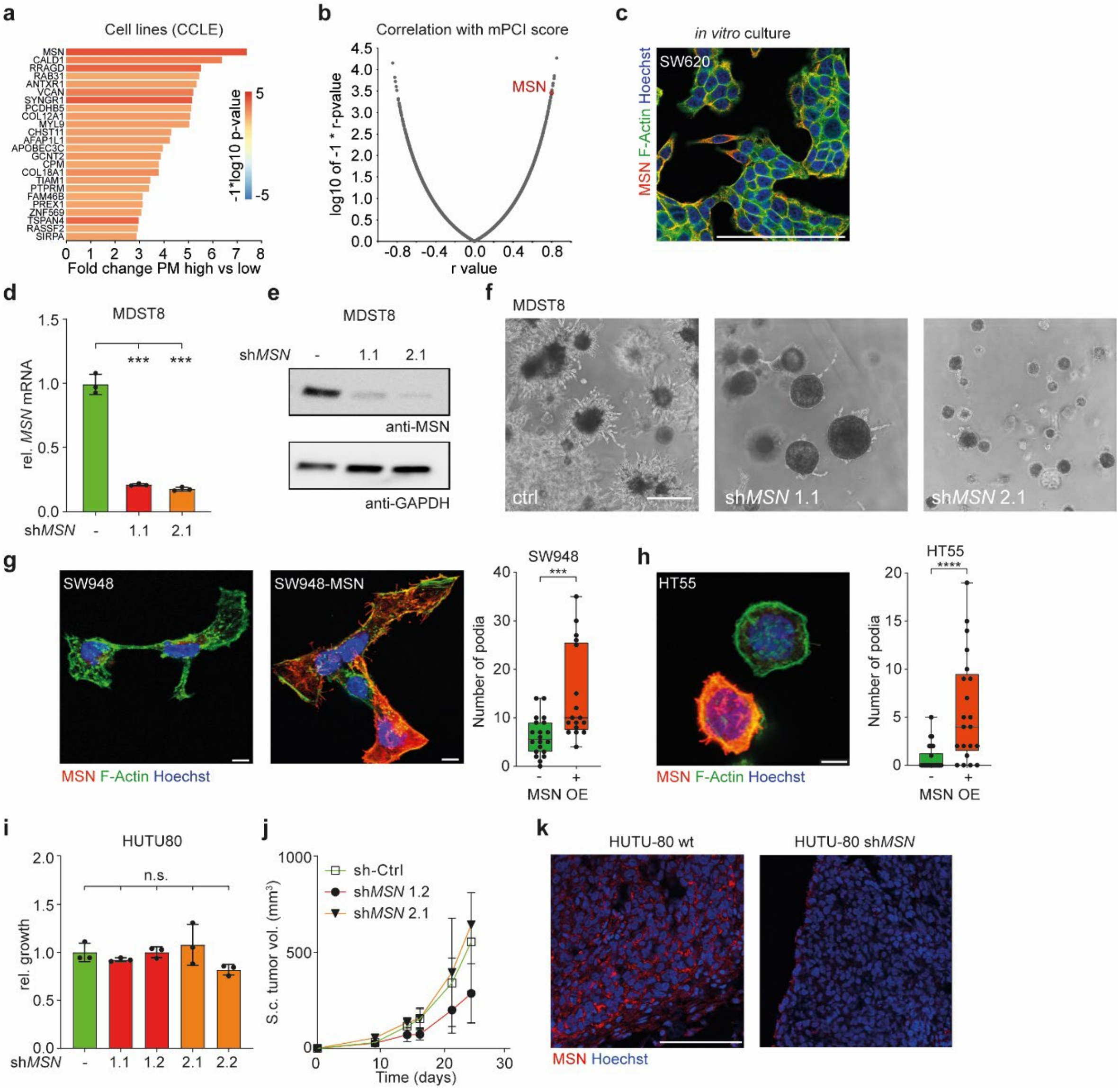

Supplementary Figure 3. *MSN* expression in peritoneal metastases. a Top 25 most differentially expressed genes in PM high CRC cell lines. **b** Correlation between gene expression and PCI score of CRC cell lines in *in vivo* PM model. **c** MSN expression in *in vitro* cultured SW620 cells, MSN (red), F-Actin (green) and nuclear staining (Hoechst, blue). Scale bars, 100 µm. Representative images of 3 independent experiments are shown. **d**, **e** Two different sh-RNAs targeting *MSN* were lentivirally transduced into MDST8 cells, single cell clones were established and MSN expression was analyzed on both mRNA (**d**) and protein level (**e**). *GAPDH* was used as a housekeeping gene (**d**, one-way ANOVA with Tukey's multiple comparisons test, *** $P = 0.0004$). **f** 3D matrigel outgrowth of *MSN* knockdown MDST8 cells is impaired. Scale bar, 50 µm. **g, h** Ectopic expression of *MSN* (red) in either SW948 (**g**) or HT55 (**h**) CRC cells. F-Actin (green) and nuclear staining (Hoechst, blue). Scale bars, 5 µm. Quantification of filopodia/cell are depicted on the right ($n = 20$ (SW948 Ctrl), $n = 17$ (SW948 MSN OE), $n = 46$ (HT55 Ctrl) and $n = 21$ (HT55 MSN OE) cells). *** $P = 0.001$ (**g**), **** $P < 0.0001$ (**h**). **i** Relative growth of either control or sh-*MSN* HUTU80 cells *in vitro*. ($n = 3$ independent experiments, *n.s.*, not significant, one-way ANOVA with Tukey's multiple comparisons test). **j** Subcutaneous tumor growth of control or sh-*MSN* HUTU80 cells ($n = 4$ independent tumors/cell line). **k** Immunofluorescent staining of subcutaneous tumors of either control or sh-*MSN*

HUTU80 cells. MSN (red), nuclear staining (Hoechst, blue). Scale bars, 100 µm. **d, i, j** Data is represented as mean ± standard deviation. **g, h** Box plots represent median, first and third quartiles (Q1 and Q3), whiskers extend to the furthest values. Source data are provided as a Source Data file.

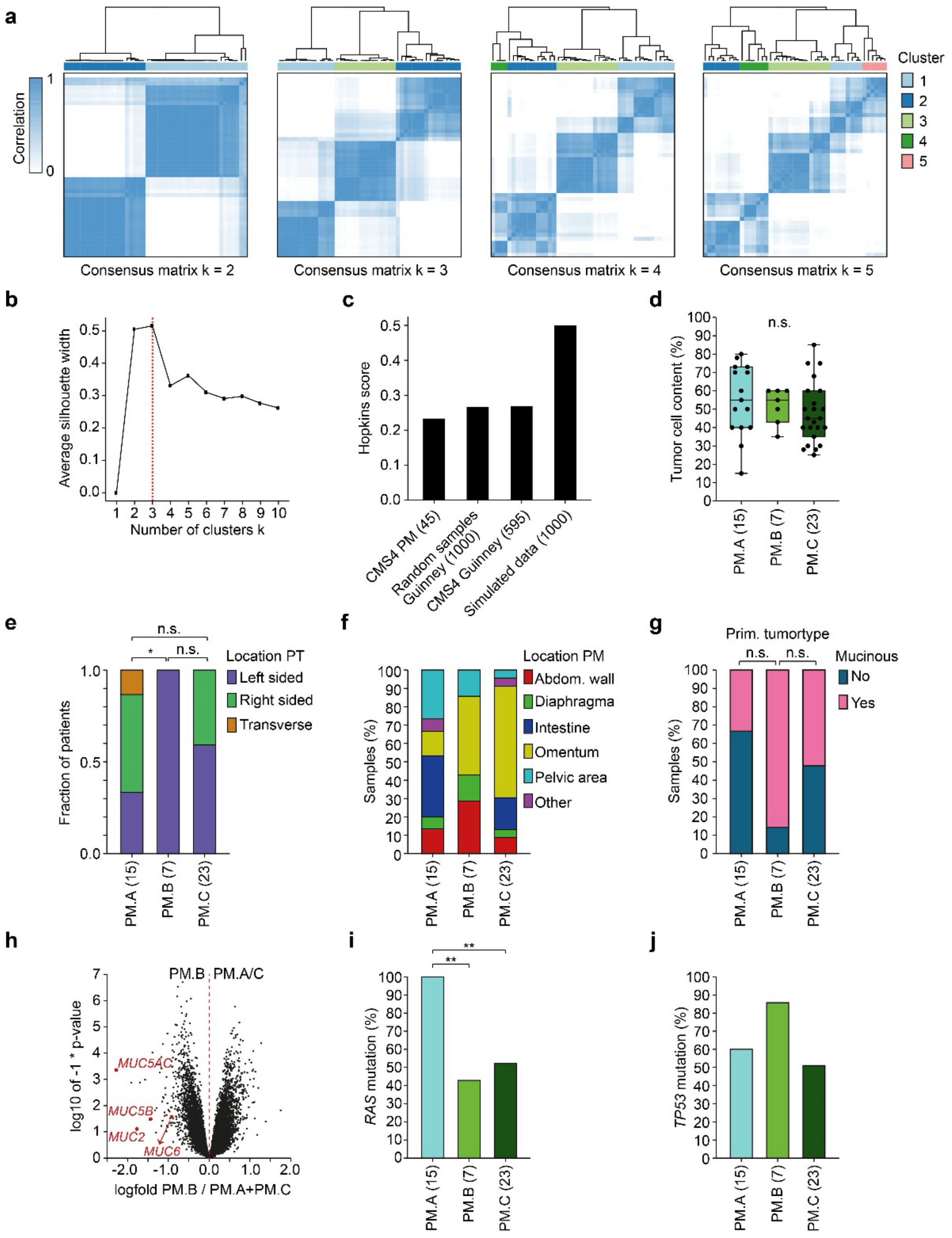

Supplementary Figure 4. CMS4-PM subgroup characterization. a Consensus matrix showing the number of potential clusters within the CMS4 PM samples (*n* = 45) for k = 2, 3, 4 and 5, using the 1,355 most differentially expressed genes. **b** Silhouette plot, using the 500 most variable genes, indicating the most optimal number of clusters (red dotted line) within the CMS4 PM samples. **c** Hopkins statistic for measuring the clustering tendency of respectively CMS4 PM dataset (*n* = 45), 1000 randomly selected CRC samples from the Guinney dataset [22], containing all 4 CMS subtypes, all CMS4 CRC samples from the Guinney dataset (*n* = 595) and a simulated dataset containing uniform data distribution (*n* = 1,000). Lower scores indicate that the dataset is cluster-tendentious. **d** Distribution of tumor cell content (%) per sample over the CMS4-PM subgroups (n = 15 (CMS4-PM.A), n = 7 (CMS4-PM.B) or n = 23 (CMS4-PM.C) biologically independent samples, one-way ANOVA, *P* = 0.420). Box plots represent median, first and third

quartiles (Q1 and Q3), whiskers extend to the furthest values. **e** Primary tumor location per CMS4-PM subgroup (Two-sided Fisher's exact test, * $P = 0.040$). **f** Peritoneal lesion location per CMS4-PM subgroup. **g** Distribution of primary tumor type over the CMS4 subgroups (Two-sided Fisher's exact test, $P = 0.058$). **h** Volcano plot depicting differentially expressed genes between subgroups PM.B versus PM.A combined with PM.C. Secreted mucins (*MUC2, MUC5AC, MUC5B* and *MUC6*) are indicated in red. **i-j** Mutation frequency of *RAS* (*KRAS* and *NRAS*) (**i**) and *TP53* (**j**) genes within the CMS4 subgroups (Two-sided Fisher's exact test, $P = 0.00109$ (*RAS*) and $P = 0.198$ (*TP53*)). Asterisks indicate level of significance: * $P < 0.05$; ** $P < 0.01$; *n.s.*, not significant. Source data are provided as a Source Data file.

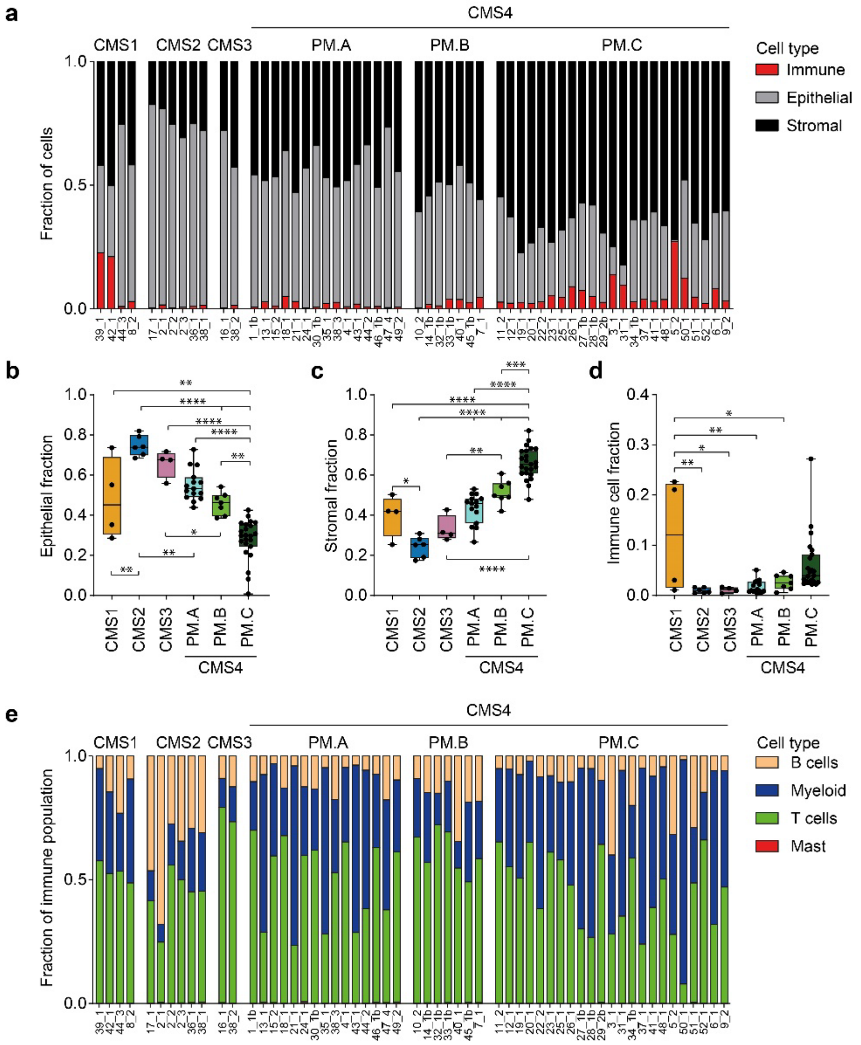

Supplementary Figure 5. Bulk RNAseq deconvolution reveals cellular composition of peritoneal metastases. a Cellular composition of all PM samples derived from deconvolution of bulk RNA seq data. **b-d** Boxplots showing distribution of epithelial (**b**), stromal (**c**) and immune cell fraction (**d**) of CMS subtyped samples (*n* = 4, 6, 4, 15, 7 and 23 independent samples for respectively CMS1, CMS2, CMS3, CMS4-PM.A, CMS4-PM.B and CMS4-PM.C). Box plots represent median, first and third quartiles (Q1 and Q3), whiskers extend to the furthest values (one-way ANOVA with Tukey's multiple comparisons test, * $P < 0.05$; ** $P < 0.01$; *** $P < 0.001$; **** $P < 0.0001$). **e** Distribution of immune cell types within immune cell fraction per sample as determined by deconvolution of bulk RNA seq data. Source data are provided as a Source Data file.

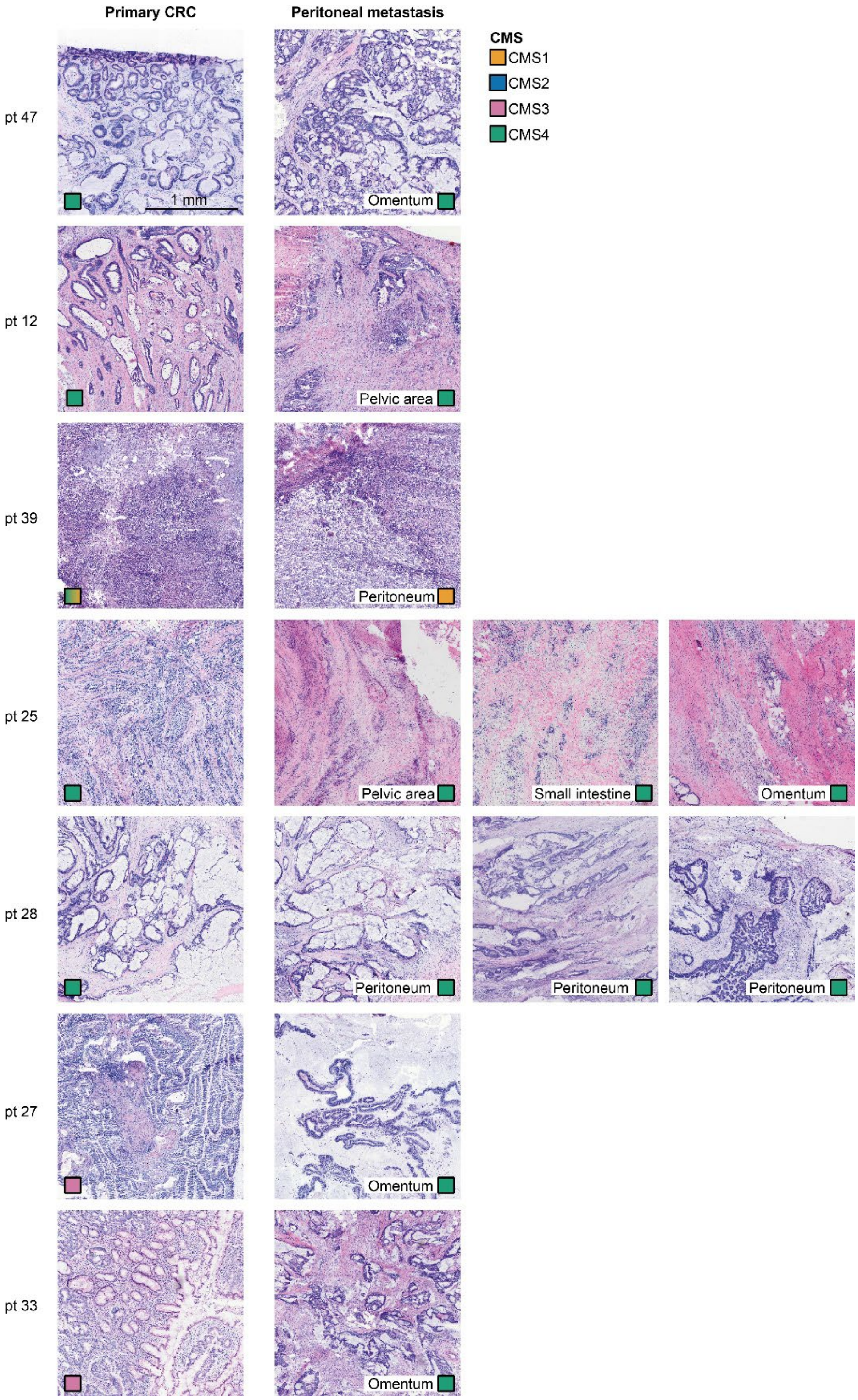

Supplementary Figure 6. Histological similarities between primary CRC and PM tissue. HE stainings of primary (PT) CRC and matching PM tissue. CMS classification is indicated with colored boxes. Anatomical location of sample is indicated. Tissue of 3 independent patients per CMS have been stained, representative images of 1 patient per CMS are shown.

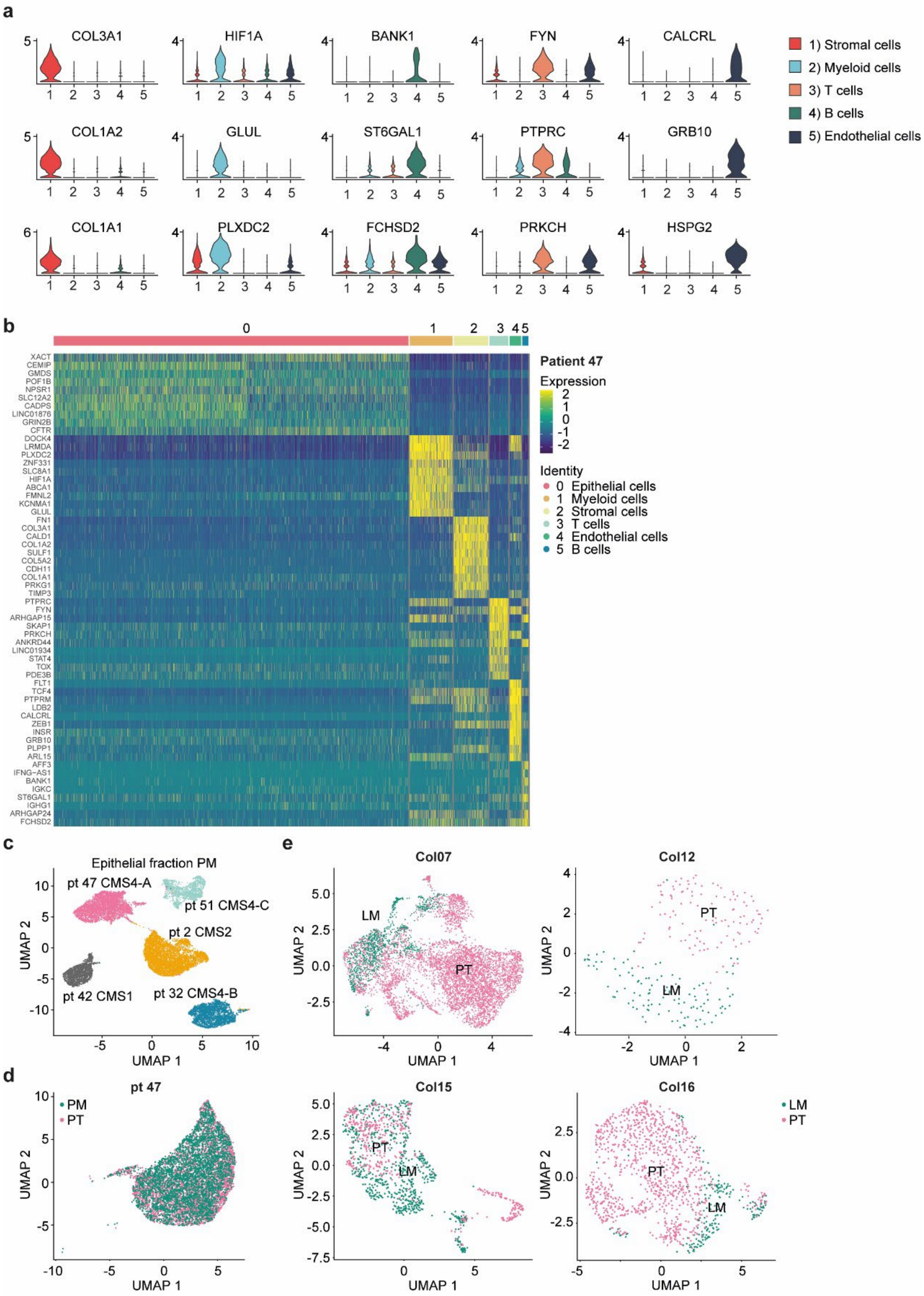

Supplementary Figure 7. Single cell RNAseq of peritoneal metastasis tissue. a Violin plots representing differentially expressed marker expression for non-epithelial cell subtypes in single cell RNA

sequenced PM tissue. **b** Heatmap showing top 10 most differentially expressed genes per cell type cluster in all cells from patient 47. **c** UMAP plot of 15,327 epithelial cells from PM samples of 5 different patients, color-coded by patient ID. **d** UMAP plots of epithelial fraction of paired PM and primary CRC (PT) (patient 47), color-coded by tissue. **e** UMAP plots of epithelial fractions of paired LM and primary CRC (PT) (Che *et al.*) samples, color-coded by tissue.

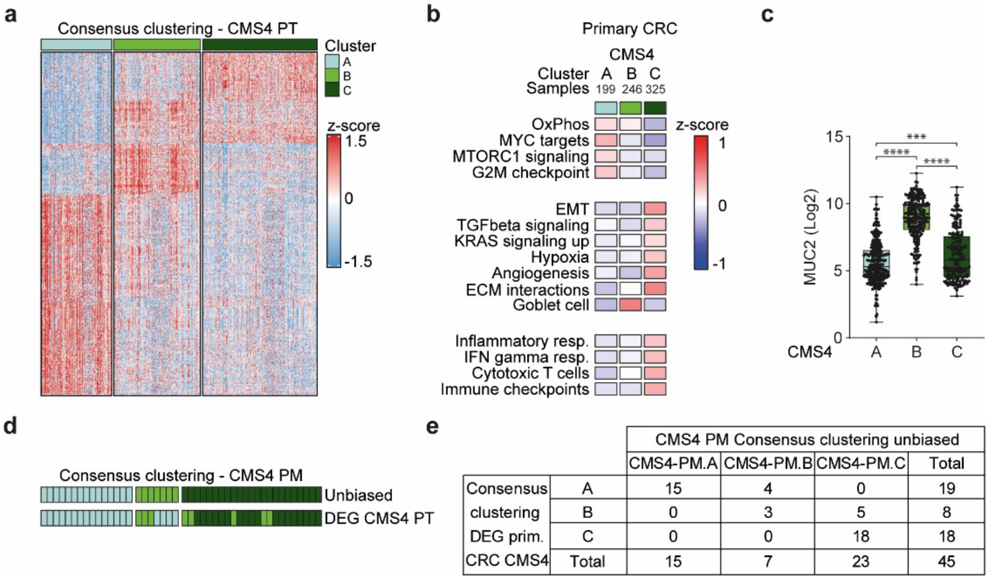

		CMS4 PM Consensus clustering unbiased			
		CMS4-PM.A	CMS4-PM.B	CMS4-PM.C	Total
Consensus	A	15	4	0	19
clustering	B	0	3	5	8
DEG prim.	C	0	0	18	18
CRC CMS4	Total	15	7	23	45

Supplementary Figure 8. Identification of CMS4 sub-clusters in primary CRCs. **a** Consensus clustering of 770 primary CRC CMS4 samples resulted in 3 subgroups with distinct gene expression. **b** Gene set enrichment analysis of CMS4 primary CRC subgroups. **c** *MUC2* expression in the 3 CMS4 clusters of primary CRCs (*n* = 325, 246 and 199 independent samples for respectively CMS4.A, CMS4.B and CMS4.C) Box plots represent median, first and third quartiles (Q1 and Q3), whiskers extend to the furthest values (one-way ANOVA with Tukey's multiple comparisons test, asterisks indicate level of significance: *** $P < 0.001$; **** $P < 0.0001$). **d** Most differential expressed genes (DEG) between the 3 CMS4 primary CRC clusters were used to perform consensus clustering on the PM CMS4 samples (n = 45) and compared to the unbiased consensus clustering as reported in **Fig. 3a**. Colors indicate the different clusters, samples are ordered by the unbiased clustering. **e** Concordance of CMS4 PM sample clustering, using either unbiased clustering or clustering based on CMS4 primary CRC subgroup DEGs. 80% of the samples were clustered in the same group for both methods. Source data are provided as a Source Data file.

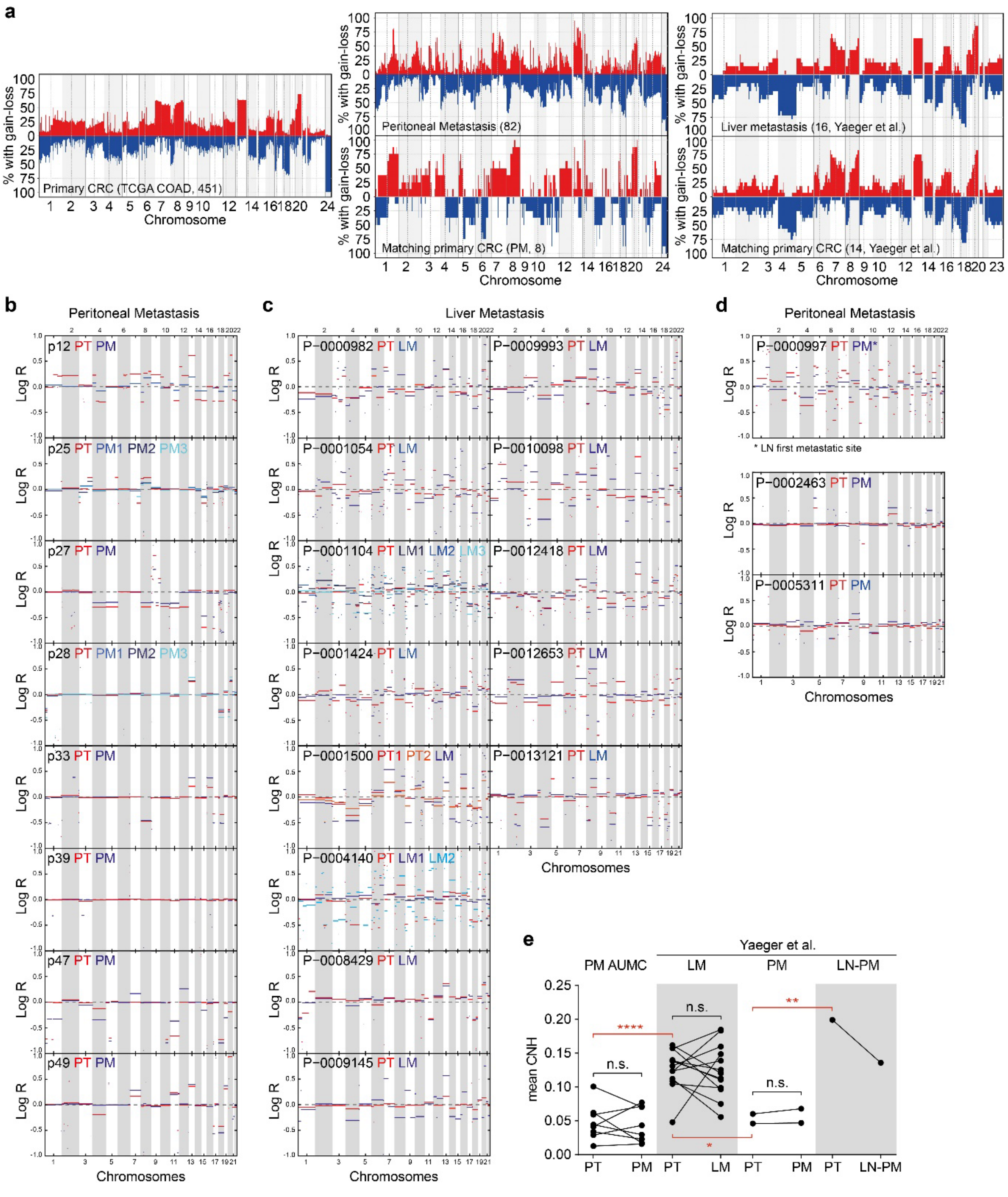

Supplementary Figure 9. Comparison of CNV profiles of primary CRC and matching metastatic tumors. a Frequency plots of DNA copy number aberrations in 451 primary CRC tumors (left panel, TCGA COAD), 82 PM samples (upper plot, middle panel) and 8 matching primary CRC samples of the PM cohort (lower plot, middle panel), 16 liver metastases (upper plot, right panel) and 14 matching primary CRC samples of the Yaeger cohort [52] (lower plot, right panel). **b** CNV profiles of primary CRC and matching PM samples of 8 patients. **c** CNV profiles of primary CRC and matching LM samples of 13 patients (Yaeger cohort). **d** CNV profiles of primary CRC and matching PM samples of 3 patients, from liver metastasis dataset (Yaeger cohort). First metastatic site of P-0000997 was in the lymph nodes (LN). **e** Copy number

heterogeneity (CNH) of primary CRC (PT) and matching LM or PM samples (one-way ANOVA with Tukey's multiple comparisons test). Asterisks indicate level of significance: **** $P < 0.0001$; ** $P < 0.0129$; * $P < 0.039$; *n.s.*, not significant. Source data are provided as a Source Data file.

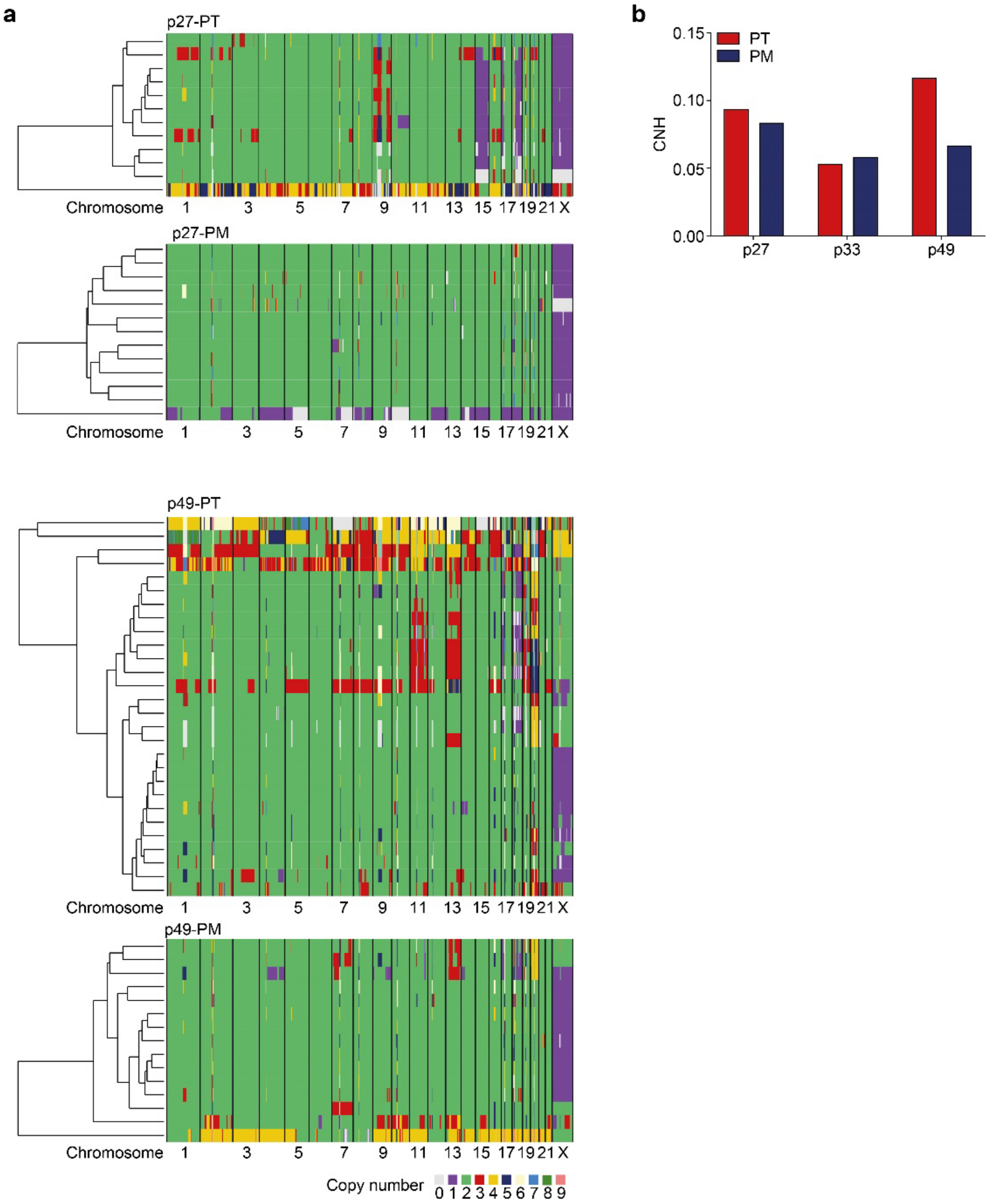

Supplementary Figure 10. Single cell CNV profiles of matching primary CRC and PM. a Heatmaps showing hierarchical clustering of single cell CNV profiles of matching primary CRC (upper) and PM samples (lower) of 2 patients. Each row represent a single cell. Colors indicate ploidy number. **b** Copy number heterogeneity (CNH) of primary CRC and matching PM samples derived from single cell CNV profiles. Source data are provided as a Source Data file.
