# Supplementary material for: Molecular characterization of colorectal cancer related peritoneal metastatic disease
Source: Nat Commun. 2022 Aug 4;13:4443. doi: 10.1038/s41467-022-32198-z (PMC9352687; doi:10.1038/s41467-022-32198-z)
Supplement: Supplementary file 2 — Reporting Summary [file 41467_2022_32198_MOESM2_ESM.pdf]

nature portfolio

Corresponding author(s): L. Vermeulen

Last updated by author(s): Jul 6, 2022

Reporting Summary

Nature Portfolio wishes to improve the reproducibility of the work that we publish. This form provides structure for consistency and transparency in reporting. For further information on Nature Portfolio policies, see our Editorial Policies and the Editorial Policy Checklist.

Statistics

For all statistical analyses, confirm that the following items are present in the figure legend, table legend, main text, or Methods section.

n/a	Confirmed	
☐	☒	The exact sample size (*n*) for each experimental group/condition, given as a discrete number and unit of measurement
☐	☒	A statement on whether measurements were taken from distinct samples or whether the same sample was measured repeatedly
☐	☒	The statistical test(s) used AND whether they are one- or two-sided<br>*Only common tests should be described solely by name; describe more complex techniques in the Methods section.*
☒	☐	A description of all covariates tested
☐	☒	A description of any assumptions or corrections, such as tests of normality and adjustment for multiple comparisons
☐	☒	A full description of the statistical parameters including central tendency (e.g. means) or other basic estimates (e.g. regression coefficient) AND variation (e.g. standard deviation) or associated estimates of uncertainty (e.g. confidence intervals)
☐	☒	For null hypothesis testing, the test statistic (e.g. *F*, *t*, *r*) with confidence intervals, effect sizes, degrees of freedom and *P* value noted<br>*Give P values as exact values whenever suitable.*
☒	☐	For Bayesian analysis, information on the choice of priors and Markov chain Monte Carlo settings
☒	☐	For hierarchical and complex designs, identification of the appropriate level for tests and full reporting of outcomes
☐	☒	Estimates of effect sizes (e.g. Cohen's *d*, Pearson's *r*), indicating how they were calculated

Our web collection on statistics for biologists contains articles on many of the points above.

Software and code

Policy information about availability of computer code

Data collection

Data for this study was collected using:

Philips Digital Pathology software
Torrent suite software v5.10.1
Leica Application Suite-Advanced Fluorescence software (v 3.5.7)

Data analysis

Data for this study was analyzed using:
CellRanger v3.1.0 software (10x Genomics)
Umap-learn v0.4.6
FastQC (Available online at: http://www.bioinformatics.babraham.ac.uk/projects/fastqc/)
RSEM v1.3.1
STAR aligner v2.6.1d
R (R Core Team, 2020)
R package org.Hs.eg.db (Genome wide annotation for Human. R package version 3.8.2.
R package tximport v1.18.0
R package 'CMSclassifier' v1.0.0
R package ConsensusClusterPlus v1.54.0
R package "Clustertend" version 1.5
ComplexHeatMap v2.6.2
RNA seq deconvolution: Deep learning based method Scaden v0.9.4
Seurat R package version 3.2.2
SeqNext software v4.1.2 (JSI Medical Systems)

MSI Analysis System, version 1.2 (Promega)
GenomeStudio 2.0 (Illumina)
Graphpad Prism 9
SPSS statistics v26 (IBM)
DNAcopy R package (version 1.58.0)
R2: Genomics Analysis and Visualization Platform (http://r2.amc.nl)

All scripts used for data analysis are available at https://github.com/vermeulenlab/peritoneal_metastasis (10.5281/zenodo.6779129)

For manuscripts utilizing custom algorithms or software that are central to the research but not yet described in published literature, software must be made available to editors and reviewers. We strongly encourage code deposition in a community repository (e.g. GitHub). See the Nature Portfolio guidelines for submitting code & software for further information.

Data

Policy information about availability of data

All manuscripts must include a data availability statement. This statement should provide the following information, where applicable:

- Accession codes, unique identifiers, or web links for publicly available datasets
- A description of any restrictions on data availability
- For clinical datasets or third party data, please ensure that the statement adheres to our policy

The sequence libraries generated in this study are publicly available through the National Center for Biotechnology Information (NCBI) Gene Expression Omnibus (GEO) under accession code: GSE183202 [https://www.ncbi.nlm.nih.gov/bioproject/?term=GSE183202]. Single-cell karyotypes of PM samples are available from EGA, accession number EGAS00001004702 [https://ega-archive.org/studies/EGAS00001004702]. Shallow sequencing data of matching primary CRC and peritoneal metastasis samples are available from SRA, accession number PRJNA841870 [https://www.ncbi.nlm.nih.gov/bioproject/PRJNA841870]. Other datasets used in this study are publicly available under accession numbers: GSE36133 [https://www.ncbi.nlm.nih.gov/bioproject/?term=GSE36133], GSE33113 [https://www.ncbi.nlm.nih.gov/bioproject/?term=GSE33113], EGAS00001002197 [https://ega-archive.org/studies/EGAS00001002197], GSE50760 [https://www.ncbi.nlm.nih.gov/bioproject/?term=GSE50760], GSE132465 [https://www.ncbi.nlm.nih.gov/bioproject/?term=GSE132465], GSE178318 [https://www.ncbi.nlm.nih.gov/geo/query/acc.cgi?acc=GSE178318], SRP029880 [https://www.ncbi.nlm.nih.gov/geo/query/acc.cgi?acc=GSE50760], http://gdac.broadinstitute.org/ for TCGA COAD, and Synapse, syn2623706 [https://www.synapse.org/#!Synapse:syn2623706/wiki/67246] for the Guinney dataset or through the R2: Genomics Analysis and Visualization Platform (http://r2.amc.nl). The remaining data are available within the Article, Supplementary Information or Source Data file.

Field-specific reporting

Please select the one below that is the best fit for your research. If you are not sure, read the appropriate sections before making your selection.

☒ Life sciences ☐ Behavioural & social sciences ☐ Ecological, evolutionary & environmental sciences

For a reference copy of the document with all sections, see nature.com/documents/nr-reporting-summary-flat.pdf

Life sciences study design

All studies must disclose on these points even when the disclosure is negative.

Sample size	For patient samples, the sample size was determined by the availability of tissue samples with sufficient tumor cell content and RNA quality as described below. For in vivo experiments, sample size was determined based on previous experiments with the in vivo PM mouse model (Bastiaenen et al, Lab. Invest. 2020) using power calculations. For in vitro experiments, no sample size was predetermined, but were chosen empirically based upon preliminary experiments to achieve statistical significance.
Data exclusions	All peritoneal metastasis tissue samples with an estimated mean cancer cell content above 30% were included for isolation, only RNA samples with an RIN (RNA integrity number) > 6.7 were subjected to further analysis. No other data was excluded from analysis.
Replication	The characterization of the PM patient cohort is observatory and was not replicated in another dataset.<br>All experimental data was reproduced by either biological replicates or independent technical replicates at least 3 times.
Randomization	For in vivo experiments, mice were randomly assigned to an experimental group. No randomization was done for allocation of patient material into groups, since we applied transcriptomic classification to subdivide the patients into groups. In vitro experiments were not randomized since the experiments were performed in well-controlled conditions.
Blinding	For all in vivo experiments, peritoneal outgrowth was scored blindly. For classification of patient material, RNA sequencing data was used and the classification is performed in an automated way. Furthermore, analysis of the patient data is descriptive and therefore blinding is not required.

Reporting for specific materials, systems and methods

We require information from authors about some types of materials, experimental systems and methods used in many studies. Here, indicate whether each material, system or method listed is relevant to your study. If you are not sure if a list item applies to your research, read the appropriate section before selecting a response.

Materials & experimental systems

n/a	Involved in the study	
☐	☒	Antibodies
☐	☒	Eukaryotic cell lines
☒	☐	Palaeontology and archaeology
☐	☒	Animals and other organisms
☐	☒	Human research participants
☒	☐	Clinical data
☒	☐	Dual use research of concern

Methods

n/a	Involved in the study	
☒	☐	ChIP-seq
☒	☐	Flow cytometry
☒	☐	MRI-based neuroimaging

Antibodies

Antibodies used	Western blotting:<br>rabbit anti-MSN (HPA011135, Sigma, 1:100)<br>mouse anti-GAPDH (MAB374/6C5, Merck, 1:1000)<br>HRP goat-anti-rabbit IgG (4050-05, Southern Biotech, 1:10.000)<br>HRP goat-anti-mouse IgG (1031-05, Southern Biotech, 1:10.000)<br><br>Immunofluorescent imaging:<br>rabbit anti-MSN (HPA011135, Sigma, 1:100)<br>goat-anti-rabbit-Alexa546 (A11035, Invitrogen, 1:500)
Validation	All antibodies used in this study are commercially available, and product information is readily available at the manufacturer's website. Validation data for all used applications for the rabbit anti-MSN antibody (HPA011135, Sigma) is available at the Human Protein Atlas website (https://www.proteinatlas.org/ENSG00000147065-MSN/antibody).<br>Anti-mouse GAPDH was used as a loading control for western blot. This antibody has been validated for western blot by the manufacturer and shows reactivity in human, mouse, canine, rat, rabbit, fish, feline and pig samples.

Eukaryotic cell lines

Policy information about cell lines

Cell line source(s)	Cell lines T84 (ATCC), SW48 (ATCC), HT55 (ECACC), SW948 (ATCC), LS180 (ATCC), HUTU80 (ATCC), SW620 (NCI), OUMS-23 (JCRB), HCT116 (NCI), KM12 (NCI), LS411N (ATCC), SNU-C1 (ATCC), LS513 (ATCC), MDST8 (ECACC) and NCI-H716 (ATCC) were obtained from the Sanger Institute (Cambridge, UK). Oroginal provider is indicated between brackets.
Authentication	All cell lines have been authenticated by STR Genotyping.
Mycoplasma contamination	All cell lines were checked for mycoplasm contamination on a monthly basis, all used cell lines tested negative.
Commonly misidentified lines<br>(See ICLAC register)	This study did not made use of commonly misidentified lines.

Animals and other organisms

Policy information about studies involving animals; ARRIVE guidelines recommended for reporting animal research

Laboratory animals	A-thymic nude mice (Hsd:Athymic Nude-Fox1nu), female, 6-12 weeks old at the start of the experiment. The mice were housed on a 12h light-dark cycle at 20-26°C with 30-70% humidity.
Wild animals	The study did not involve wild animals
Field-collected samples	This study did not involve samples collected from the field.
Ethics oversight	All animal experiments were approved by the Animal Experimentation Committee at the Academic Medical Center in Amsterdam (AVD118002016493) and conducted in accordance with the national guidelines.

Note that full information on the approval of the study protocol must also be provided in the manuscript.

Human research participants

Policy information about studies involving human research participants

Population characteristics	In this study we used tumour samples from 52 patients (male and female) with peritoneal metastasis, collected at the Amsterdam University Medical Center, location VUmc between 2010-2018. Eligibility criteria for inclusion were: histologically proven colorectal carcinoma with synchronous or metachronous peritoneal metastasis, age older than 18 years and fresh

	frozen tissue available. The detailed clinical and histopathological characteristics of this cohort are described in Supplementary Table 1.
Recruitment	No patients have been recruited for this study, we used available material.
Ethics oversight	Patient samples were collected according to Dutch research guidelines of the Federation of Dutch Medical Scientific Societies (FDMSS), as described in "Human Tissue and Medical Research: Code of Conduct for Responsible use". When required, patients provided informed consent for sampling additional tumor tissue for study purposes.

Note that full information on the approval of the study protocol must also be provided in the manuscript.
